# Preferential consolidation of emotional reactivity during sleep: A systematic review and meta-analysis

**DOI:** 10.3389/fnbeh.2022.976047

**Published:** 2022-10-04

**Authors:** Gosia Lipinska, Holly Austin, Jasmin R. Moonsamy, Michelle Henry, Raphaella Lewis, David S. Baldwin, Kevin G. F. Thomas, Beth Stuart

**Affiliations:** ^1^UCT Sleep Sciences and Applied Cognitive Science and Experimental Neuroscience Team (ACSENT), Department of Psychology, University of Cape Town, Cape Town, South Africa; ^2^Clinical and Experimental Sciences, Faculty of Medicine, University of Southampton, Southampton, United Kingdom; ^3^Numeracy Centre, Centre for Higher Education Development, University of Cape Town, Cape Town, South Africa; ^4^Department of Psychiatry and Mental Health, University of Cape Town, Cape Town, South Africa; ^5^Centre for Evaluation and Methods, Wolfson Institute of Population Health, Queen Mary University of London, London, United Kingdom

**Keywords:** sleep, emotion, emotional reactivity, emotional regulation, meta-analysis, review, consolidation

## Abstract

Many studies have investigated whether sleep affects cognitively unmodulated reactivity to emotional stimuli. These studies operationalize emotion regulation by using subjective and/or objective measures to compare pre- and post-sleep reactivity to the same emotional stimuli. Findings have been inconsistent: some show that sleep attenuates emotional reactivity, whereas others report enhanced or maintained reactivity. Across-study methodological differences may account for discrepant findings. To resolve the questions of whether sleep leads to the attenuation, enhancement, or maintenance of emotional reactivity, and under which experimental conditions particular effects are observed, we undertook a synthesized narrative and meta-analytic approach. We searched PubMed, PsycINFO, PsycARTICLES, Web of Science, and Cochrane Library databases for relevant articles, using search terms determined *a priori* and search limits of language = English, participants = human, and dates = January 2006–June 2021. Our final sample included 24 studies that investigated changes in emotional reactivity in response to negatively and/or positively valenced material compared to neutral material over a period of sleep compared to a matched period of waking. Primary analyses used random effects modeling to investigate whether sleep preferentially modulates reactivity in response to emotional stimuli; secondary analyses examined potential moderators of the effect. Results showed that sleep (or equivalent periods of wakefulness) did not significantly affect psychophysiological measures of reactivity to negative or neutral stimuli. However, self-reported arousal ratings of negative stimuli were significantly increased post-sleep but not post-waking. Sub-group analyses indicated that (a) sleep-deprived participants, compared to those who slept or who experienced daytime waking, reacted more strongly and negatively in response to positive stimuli; (b) nap-exposed participants, compared to those who remained awake or who slept a full night, rated negative pictures less negatively; and (c) participants who did not obtain substantial REM sleep, compared to those who did and those exposed to waking conditions, had attenuated reactivity to neutral stimuli. We conclude that sleep may affect emotional reactivity, but that studies need more consistency in methodology, commitment to collecting both psychophysiological and self-report measures, and should report REM sleep parameters. Using these methodological principles would promote a better understanding of under which conditions particular effects are observed.

## Introduction

Numerous investigations have examined the role of sleep in emotion regulation (i.e., whether engaging in a period of sleep, be it across a full night, a partial night, or a daytime nap, can change emotional reactions to previously encountered events or stimuli). Among these investigations are studies of pre- to post-sleep changes in mood, in cognitively modulated emotion regulation (i.e., active control of responses to emotional stimuli), and in spontaneous reactivity to emotional stimuli (for a review, see Palmer and Alfano, 2017). These studies suggest that (a) mood is significantly negatively altered (i.e., people feel more depressed, anxious, angry, and/or confused) by periods of sleep loss; (b) these feelings are amplified as the sleep deprivation period increases; (c) participants with mood- and anxiety-related clinical symptoms who sleep poorly are less likely to use cognitive reappraisal strategies to modulate the negative impact of emotional experiences (see, e.g., Mauss et al., 2013; Baum et al., 2014; Short and Louca, 2015); and (d) participants who are sleep deprived, either experimentally or because of a sleep disorder (e.g., insomnia), do not extinguish reactivity to a previously conditioned stimulus after a period of sleep (Seo et al., 2018, 2022; Bottary et al., 2020). Regarding this latter point, although extinction is considered a form of emotional learning, successful extinction leads to an emotionally regulated state, where the individual is not likely to react inappropriately to unthreatening negative cues in the environment (Picó-Pérez et al., 2019; Frumento et al., 2021).

However, a larger group of studies within the sleep-emotion regulation literature has examined whether sleep affects spontaneous reactivity to emotional stimuli (i.e., reactivity that is not modulated through explicit or active use of emotion regulation strategies). The typical paradigm in this literature operationalizes emotional change by comparing pre- and post-sleep reactivity to the same set of emotional stimuli. This reactivity may be measured using either subjective or objective measures, with the latter including psychophysiological outcomes such as skin conductance levels and heart rate-associated variables (e.g., heart rate deceleration).

The outcome in these studies is the degree to which participants react differently to the same stimuli after a period of sleep as compared to an equivalent period of waking. An attenuated response (rather than an enhanced or maintained response) is considered more adaptive because in healthy individuals repeated exposure to emotional (and particularly negatively valenced) stimuli during waking hours and in an unthreatening environment is associated with increasingly attenuated responses to the material (Minkel et al., 2011; Baran et al., 2012). In this way, hyperactivation of fear networks that are associated with pathology (e.g., posttraumatic stress disorder) is avoided.

However, the exact mechanisms underlying modulation of emotional reactivity during sleep are not well understood. Neuroimaging studies show that regions involved in emotional processing, such as the anterior cingulate cortex, hippocampus, parahippocampus, amygdalar complex, pontine tegmentum, thalamus, and basal forebrain are active during sleep, and especially during rapid eye movement (REM) stages (Dang-Vu et al., 2010). Furthermore, a small number of studies show that after exposure to emotional material, brain regions associated with those stimuli (hippocampus and ventral tegmental area) are activated during sleep; notably, the magnitude of their activation is correlated with post-sleep task performance (Sterpenich et al., 2021; Legendre et al., 2022). These findings suggest that there is selective replay of emotional content during sleep, and that this replay aids in consolidation of emotional learning.

Limited understanding of mechanisms underlying the sleep-emotion regulation relations stands alongside inconsistent findings from studies investigating these relations. Whereas some studies show that sleep does attenuate reactivity to emotional stimuli (Gujar et al., 2011; Palmer and Alfano, 2017), others report enhanced (Wagner et al., 2002; Gilson et al., 2016; Jones et al., 2018) or maintained (Baran et al., 2012; Prehn-Kristensen et al., 2017) reactivity. For example, Cellini et al. (2016) found that a nap, compared to an equivalent period of waking, attenuated self-reported negative affect in response to negatively valenced (but not neutral) stimuli. However, Jones et al. (2018) found that, after a night of sleep in comparison to a day of wakefulness, participants tended to have elevated self-reported negative affect in response to negatively valenced (but not neutral) stimuli. Furthermore, Prehn-Kristensen et al. (2017) reported no significant differences in both post-sleep and post-waking reactivity to all stimuli (emotionally valenced and neutral).

Differences in methodology may account for these across-study discrepancies in findings. These methodological differences include variations in the timing and duration of the sleep condition, whether participants obtained REM sleep (this stage of sleep appears to be central to the emotional regulatory benefits of sleep; Gujar et al., 2011; Palagini et al., 2013; Deliens et al., 2014; Altena et al., 2016), the type of waking control used, the kind of emotional stimuli presented, and the primary outcome measure used. However, because sleep can have clear benefits for emotion processing and because spontaneous and unmodulated emotional reactivity is an important influence on human behavior (Levenson, 1999; Gross et al., 2011; Becerra and Campitelli, 2013; Palmer and Alfano, 2017), it is important to determine whether a discernible pattern of sleep-dependent emotional regulation might emerge from this seemingly equivocal literature.

## The current study

The existing literature in this field has not clearly answered the questions of (a) whether sleep leads to attenuation, enhancement, or maintenance of emotional reactivity, and (b) under which experimental conditions particular directions of results are observed. We conducted a systematic review with a narrative synthesis and meta-analysis, reviewing 24 studies that reported emotional reactivity for negatively and positively valenced material compared to neutral material over any period of sleep (whole night or nap) compared to a matched period of waking or sleep deprivation (i.e., wakefulness during either the day or the night). After a series of primary analyses assessing the general question of whether sleep preferentially modulates emotional reactivity in response to emotional stimuli, secondary analyses examined potential moderators of the effect.

## Methods

### Systematic review protocol

The study protocol was submitted and approved for registration on PROSPERO: https://www.crd.york.ac.uk/prospero/display_record.php?RecordID=271030.

### Search strategy

**Figure 1:**
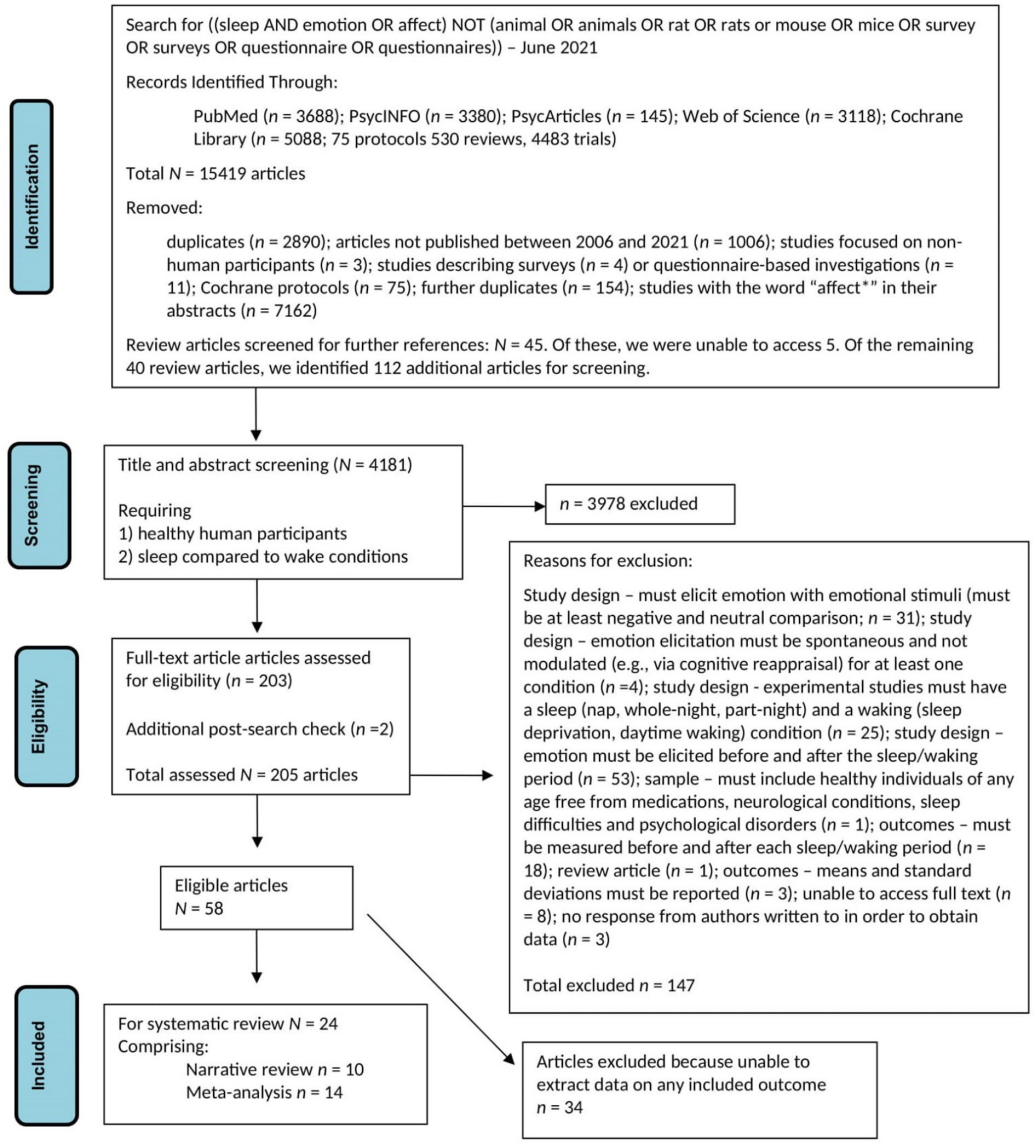
Flow diagram for literature search and subsequent screening and evaluation of retrieved articles.

Figure 1 is a PRISMA diagram providing details of the search process and of how we arrived at our final sample of articles for review.

Two authors (GL, JM) searched PubMed, PsycINFO, PsycARTICLES, Web of Science, and Cochrane Library databases using these terms: [(*sleep* AND *emotion* OR *affect*) NOT (*animal* OR *animals* OR *rat* OR *rats* OR *mouse* OR *mice* OR *survey* OR *surveys* OR *questionnaire* OR *questionnaires*)]. These terms were determined *a priori*, and the search was limited to articles published in English between January 1st 2006 and June 2nd 2021. The search process continued until July 2021. A total of 15,419 articles were retrieved.

GL and JM reviewed this dataset and removed duplicates, Cochrane protocols, and those not meeting the specified criteria. The term “affect” was excluded as it resulted in retrieval of articles that featured that word in the abstract, but which were unrelated to our research topic (e.g., how particular environments might affect animals' adaptive abilities). This initial scan of the dataset identified 45 review articles that might have been useful in alerting us to empirical articles of interest. Hence, author HA screened the full text of those review articles that were accessible (*n* = 40): a process which identified a further 112 articles.

At that point, a total of 4,181 articles remained for title and abstract screening. Three pairs of authors (JRM, KGFT; RL, DSB; GL, MH) each screened one-third of the papers, with each pair holding consensus meetings to finalize inclusion/exclusion decisions for each article. These decisions rested largely on whether the title/abstract made it clear that this was a study that (1) included healthy human participants and (2) described an experiment that compared a sleep condition to a waking control condition. As a result, 3,978 papers were excluded, leaving 203 articles for full-text screening. A further two papers were identified at an additional post-search check, resulting in a sample of 205 articles put forward for full-text screening.

The same author pairs then undertook detailed reviews of the full-text articles. They organized their examination of each paper so that they could make decisions around six specific eligibility criteria. These criteria were that the study must have:

1. used emotion-eliciting stimuli, and must have included at least a comparison of negatively valenced vs. neutral material;
2. elicited emotion spontaneously and not *via* modulated means (e.g., *via* cognitive appraisal);
3. been an experiment featuring at least one sleep condition (nap, whole night, or partial night) and at least one waking condition (sleep deprivation or daytime waking);
4. elicited emotion both before and after a period of sleep and a period of waking;
5. featured a sample of healthy individuals of any age and free from medications, neurological conditions, sleep difficulties, or psychological disorders;
6. reported outcome measures from before and after each interval period (sleep and waking).

Any article that did not meet any one of these six criteria was excluded from further consideration. We also excluded review articles and articles that did not present complete enough information for us to assess whether they should be included in the final sample. (In cases where articles did not provide such complete information, we contacted corresponding authors for further details and further evaluated the study only if further details were forthcoming.) Eventually, our author pairs achieved consensus for each article; if there was an initial disagreement this was discussed in a group meeting and consensus reached. A total of 147 articles were excluded at this stage.

A total of 58 articles remained eligible for inclusion in the review after full-text screening and consensus meetings were completed (i.e., 147 articles were excluded at this stage). However, on further review 34 of this pool of 58 were found to report only outcome measures that were not of interest to us (e.g., retention of memory for emotional vs. neutral material) or to include no extractable data for any reported outcome. A further 10 articles were only eligible for narrative systematic review of results. Of the remaining 14 articles, we were able to extract relevant statistical data directly from either text or tables in eight cases. In the other six articles, we used the WebPlotDigitizer software application (Version 4.5; Rohatgi, 2021) to extract relevant statistical data from figures.

Hence, our final sample for review was 24 studies, with 10 included only in the narrative review and 14 in meta-analysis (see Supplementary material for a brief description of each of these studies).

#### Data extraction and coding

We extracted the following sets of basic data from each of the 24 studies in our sample: study design (between-subjects or crossover); type of sleep condition (whole night or nap); type of comparison condition (wake or sleep deprivation); emotion elicitation technique [e.g., International Affective Picture System (IAPS), in-house pictures, Nencki Affective Picture System (NAPS), Ekman library of pictures, movie clips, emotional faces]; whether or not IAPS stimuli were used; total number of participants enrolled in the study; total number of participants who completed the protocol; sample age (*M*); and number of female participants (see Tables 1–4).

**Table 1:** Datasets included in the meta-analysis: Study design, conditions, and sample characteristics (*N* = 14).

**Study/Dataset**	**Sleep condition**		**Waking comparison condition**		**Sample characteristics**	
	**Type**	***n***	**Type**	***n***	**Age range (years)**	**Sex**
1. Alfarra et al. (2015)	Full night	10	Full day	10	18–30	Mixed
2. Ashton et al. (2019)	Full night	34	Full day	27	NR	Mixed
3. Baran et al. (2012)	Full night	54	Full day	28	18–30	Mixed
4. Bolinger et al. (2018)	Full night (10)	16	Full day (10)	16	8–11	Mixed
5. Bolinger et al. (2019)	Full night (10)	16	Full day (10)	16	19–29	Mixed
6. Cellini et al. (2016)	90–120-min nap	30	90–120-min waking	16	20–30	Mixed
7. Gujar et al. (2011)	90-min nap	18	90-min waking	18	18–30	Mixed
8a. Jones et al. (2018)	Full night	20	Full day	20	18–30	Mixed
8b. Jones et al. (2018)	Full night	21	Full day	20	35–50	Mixed
9. Kuriyama et al. (2010)	Full night	14	Sleep deprivation	14	20–33	Mixed
10. Lipinska and Thomas (2019)	Full night	20	Full day	20	NR	All female
11. Pace-Schott et al. (2011)	120-min nap	22	120-min waking	21	18–27	Mixed
12. Prehn-Kristensen et al. (2017)	Full night	16	Full day	16	9–11	All male
13. Tempesta et al. (2010)	Full night	20	Sleep deprivation	20	20–36	All female
14. Tempesta et al. (2015)	Full night	52	Sleep deprivation	23	NR	Mixed

Most studies used a between-subjects design. Only these used a crossover design: Alfarra et al. (2015), Prehn-Kristensen et al. (2017), Bolinger et al. (2018), Lipinska and Thomas (2019).

NR, not reported.

**Table 2:** Datasets included in the meta-analysis: Study stimulus characteristics and outcomes (*N* = 14).

**Study/Dataset**	**Stimulus characteristics**		**Outcome measure(s)**
	**Type**	**Valence^1^**	
1. Alfarra et al. (2015)	IAPS	–/+	Physiological: LPP/salivary cortisol
2. Ashton et al. (2019)	IAPS	–	Physiological: HRD/SCR
3. Baran et al. (2012)	IAPS	–	Self-report: Δ valence/Δ arousal
4. Bolinger et al. (2018)	IAPS	–	Physiological: LPP/HRD
5. Bolinger et al. (2019)	IAPS	–/+	Physiological: LPP/HRD
6. Cellini et al. (2016)	IAPS	–/+	Behavioral: *d'*
7. Gujar et al. (2011)	Facial expressions^2^	–/+	Behavioral: Δ emotional reactivity^3^
8a. Jones et al. (2018)	IAPS	–/+	Behavioral: *d'*
8b. Jones et al. (2018)	IAPS	–/+	Behavioral: *d'*
9. Kuriyama et al. (2010)	Movies^4^	–	Physiological and behavioral: SCR/recognition/fear rating
10. Lipinska and Thomas (2019)	IAPS	–	Physiological: HR/SCL
11. Pace-Schott et al. (2011)	IAPS	–	Physiological: SCR/HRD/EMG
12. Prehn-Kristensen et al. (2017)	Faces^5^	–/+^6^	Behavioral: *d'/*pupil reaction^7^
13. Tempesta et al. (2010)	IAPS	–/+	Self-report: Δ arousal^8^
14. Tempesta et al. (2015)	IAPS	–/+	Self-report: Δ valence/Δ arousal

IAPS, International Affective Picture System; LPP, late positive potential of the electroencephalogram (EEG); HRD, heart rate deceleration (emotional response to hits at recognition minus emotional response to same stimuli at encoding); SCR, skin conductance response; HR, heart rate; SCL, skin conductance level; EMG, electromyography.

d' (d prime) measures memory discrimination, and is calculated as z(Hit Rate) – z(False Alarm Rate).

^1^Studies presented the following variations of valence-based analyses: –na = negative and positive stimuli presented and analyzed separately; – = negative stimuli only presented and analyzed.

^2^Ekman pictures of facial affect (fearful, sad, angry, happy).

^3^Δ emotional reactivity = change from pre- to post-manipulation in the rating for each of the individual faces in a specified emotional category.

^4^14 movies, 7 of which showed safe driving and 7 of which showed a motor vehicle accident, accompanied by realistic sounds.

^5^320 black and white pictures of faces showing different kinds of emotional expressions (80 angry, 80 fearful, 80 happy, 80 neutral). Pictures were taken from the following databases: FACES (Ebner et al., 2010), Nim Stimset of Facial Expressions (Tottenham et al., 2009), 3D Facial Emotional Stimuli (Gur et al., 2002), Karolinska Directed Emotional Faces Systems (KDEF; Goeleven et al., 2008), and Productive Aging Laboratory Face Database (Minear and Park, 2004).

^6^Fearful (negative) and happy (positive) stimuli only; presented in separate blocks or trials.

^7^Binocular eye movements and pupil diameter - each event class (old/new by emotion) was averaged over the left and right eye.

^8^Valence data were collected but were not extractable.

**Table 3:** Datasets included in the narrative analysis: Study design, conditions, and sample characteristics (*N* = 10).

**Study/Dataset**	**Sleep condition**		**Waking comparison condition**		**Sample characteristics**	
	**Type**	***n***	**Type**	***n***	**Age range (years)**	**Sex**
1. Cunningham et al. (2014)	Full night	18	Full day	21	NR	Mixed
2. Goldstein et al. (2013)	Full night	18	Other	18	NR	Mixed
3. Hot et al. (2016)^1^	Nap	30	No nap	30	NR	NR
4. Kuriyama et al. (2013)	Full night	31	Sleep deprivation	31	20–19	Mixed
5. Lau et al. (2020)	Nap	19/22	No nap	25	16–60	NR
6. Minkel et al. (2011)	Full night	8	Sleep deprivation	15	22–45	Mixed
7. Reddy et al. (2014)^1^	Full night	NR	Sleep deprivation	NR	13–17	NR
8. Reid et al. (2019)^1^	Full night	24	Forced awakenings	27	NR	Mixed
9a. Schoch et al. (2017)	Full night	29	Full day	28	18–35	Mixed
9b. Schoch et al. (2017)	Full night	28	Full day	27	18–35	Mixed
10. Wagner et al. (2002)	Full night	12	Full day	12	18–30	All male

All studies used a between-subjects design.

NR, not reported.

^1^Conference abstract – limited information available.

**Table 4:** Datasets included in the narrative analysis: Study stimulus characteristics and outcomes (*N* = 10).

**Study/Dataset**	**Stimulus characteristics**		**Outcome measure(s)**
	**Type**	**Valence^1^**	
1. Cunningham et al. (2014)	Scenes^2^	–	HRD/SCR
2. Goldstein et al. (2013)	Emotion-anticipation task	–	fMRI
3. Hot et al. (2016)^3^	Movie scene	–	HRD
4. Kuriyama et al. (2013)	Movie clips^4^	–	*d'*/*C*/ΔSCR
5. Lau et al. (2020)	Emotional faces^5^	–/+	Intensity ratings of different emotions
6. Minkel et al. (2011)	Movie clips^6^		Facial expressiveness^7^
7. Reddy et al. (2014)^3^	IAPS Pictures	–/+	Affectivity
8. Reid et al. (2019)^3^	Words^8^	–	Attentional bias index^9^
9a. Schoch et al. (2017)	IAPS Pictures	–/+	Free recall
9b. Schoch et al. (2017)	IAPS Pictures	–/+	Free recall
10. Wagner et al. (2002)	IAPS Pictures	–	Valence/arousal

HRD, heart rate deceleration; SCR, skin conductance response; fMRI, neuronal activity as measured by functional magnetic resonance imaging. d' (d prime) measures memory discrimination, and is calculated as z(Hit Rate) - z(False Alarm Rate). C is a measure of recognition bias, calculated as 0.5 × z(Hit Rate) + 0.5× z(false alarm rate).

^1^Studies presented the following variations of valence-based analyses: –/+ = negative and positive stimuli presented and analyzed separately; – = negative stimuli only presented and analyzed.

^2^Participants viewed a set of 68 scenes that portrayed negatively arousing or neutral objects (34 of each valence) placed on plausible neutral backgrounds.

^3^Conference abstract – limited information available.

^4^Clips of motor vehicle accidents and of safe driving situations.

^5^Black-and-white pictures of faces of a Caucasian male and a Caucasian female expressing four emotions (happiness, sadness, fear, anger) were selected from the Karolinska Directed Emotional Faces (KDEF) set (Goeleven et al., 2008).

^6^Participants watched two film clips that were either sad or amusing. They were then randomized to either a night of sleep deprivation or a full night of sleep before watching another pair of sad and amusing clips.

^7^Videos were scored for global level of expressiveness based on the FACES scoring system (Kring and Sloan, 2007) by two raters.

^8^Threat-related versus neutral words.

^9^The extent to which participants showed preferential attentional allocation toward threat-related vs. neutral words.

For the 14 studies included in the meta-analysis, we also extracted physiological and/or self-report emotion regulation outcome data (*M* and *SD* or *SEM*, as well as confidence intervals and *p*-values if those were available) for each stimulus category (negative, positive, and/or neutral), at each of the pre- and post-condition measurement points, for each of the sleep and comparison conditions. Physiological variables included heart rate deceleration (HRD), skin conductance response (SCR) or skin conductance level (SCL), pre-ejection period (PEP), and late positive potential (LPP) of the electroencephalogram. Self-report variables included valence and arousal ratings in response to the presented stimuli.

Finally, we coded each study's risk of bias as high, low, or unclear along the following dimensions: (a) clear definition of the study sample; (b) clear stipulation of study eligibility criteria and clear demonstration of how these were enforced; (c) clear definition of the sampling strategy/ies; (d) matching of groups/conditions on sociodemographic and/or other characteristics; (e) control of confounds (e.g., caffeine intake, daytime nap, adaptation night) for each sleep and comparison condition; (f) the quality and validity of outcome measures; (g) number of participants completing the study protocols >80%; (h) reporting on and accounting for missing data; (i) reporting of all study parameters; (j) use of WebPlotDigitizer to extract data; (k) statistical adjustment of results for confounders; and (1) other potential sources of bias.

All extracted data were entered into a Microsoft Excel spreadsheet, where they were cleaned and prepared for further analysis.

#### Narrative synthesis

Because not all study characteristics and outcomes can be described adequately in accompanying tables, we include a narrative account of those articles whose study descriptions precluded incorporation of data into the meta-analysis (*n* = 10) as well as those that were included in the meta-analysis (*n* = 14).

#### Meta-analysis

Before analysis, we completed a rigorous process of coding outcome variables in each study to ensure consistent directionality for both psychophysiological and self-report data. For example, in the case of HRD more negative beats-per-minute values represent greater emotional reactivity. Hence, attenuation over the sleep or waking interval (post-interval minus pre-interval) is described by values that are larger and positive. In contrast, for SCR larger positive values represent greater emotional reactivity and, therefore, attenuation over the sleep or waking interval is described by values that are larger and negative. We completed a similar process of study-by-study evaluation for self-report data, which also varied depending on the stimulus used (e.g., IAPS pictures, film clips, or faces) and the subsequent method of emotion measurement related to that stimulus (e.g., self-assessment manikin, other rating scale).

Due to anticipated between-study heterogeneity, we pooled studies appropriate for meta-analysis using a generic inverse variance random effects model. Because different scales were reported across different studies, we report the meta-analysis of standardized mean differences, with associated 95% confidence intervals. The standardized mean difference can be interpreted in a similar manner to a Cohen's *d* standardized effect size.

Because the studies included in our review sample reported a mix of separate baseline and follow-up measures and collapsed change from baseline to follow-up scores, it was not possible to include all types of outcomes in a standardized mean differences meta-analysis. So, to maximize the data available for analysis, we used change scores only after (where necessary) converting separate baseline and follow-up values using procedures set out in the Cochrane Handbook (Section 6.5.2.8; Version 6.3; Higgins et al., 2022), with a conservative assumed correlation between measures of 0.5.

Because we sought to undertake the relatively complex tasks of exploring both valence and condition differences in the same meta-analysis, we undertook analyses in two stages. The first involved investigation of our primary question: i.e., is emotionally valenced material regulated more strongly than neutral material (e.g., is reactivity to emotional stimuli attenuated while reactivity to neutral material is not) over a period of sleep as compared to over a similar period of waking? Hence, at this stage we calculated the standardized mean change (SMC), in terms of emotional reactivity, for (a) valenced material over a period of sleep vs. over a comparison period (i.e., daytime waking, sleep deprivation), and then (b) neutral material over a period of sleep vs. over a comparison period. We ran separate analyses for the psychophysiological (e.g., heart rate deceleration) outcomes and the self-report (valence, arousal) outcomes. In cases where it appeared there was indeed preferential regulation of emotionally valenced material, we then compared the SMC from the valenced condition(s) in the sleep-vs.-comparison analysis to the SMC from the neutral condition in the sleep-vs.-comparison analysis. The methods used for the calculations were as set out in Morris (2008) and Schäfer et al. (2020).

Funnel plots were produced to examine evidence of publication bias in all analytic comparisons that included 10 or more studies. Egger's test was carried out to test for small study effects.

We used the meta command in Stata version 17 for all analyses.

## Results

### Risk of bias

**Figure 2:**
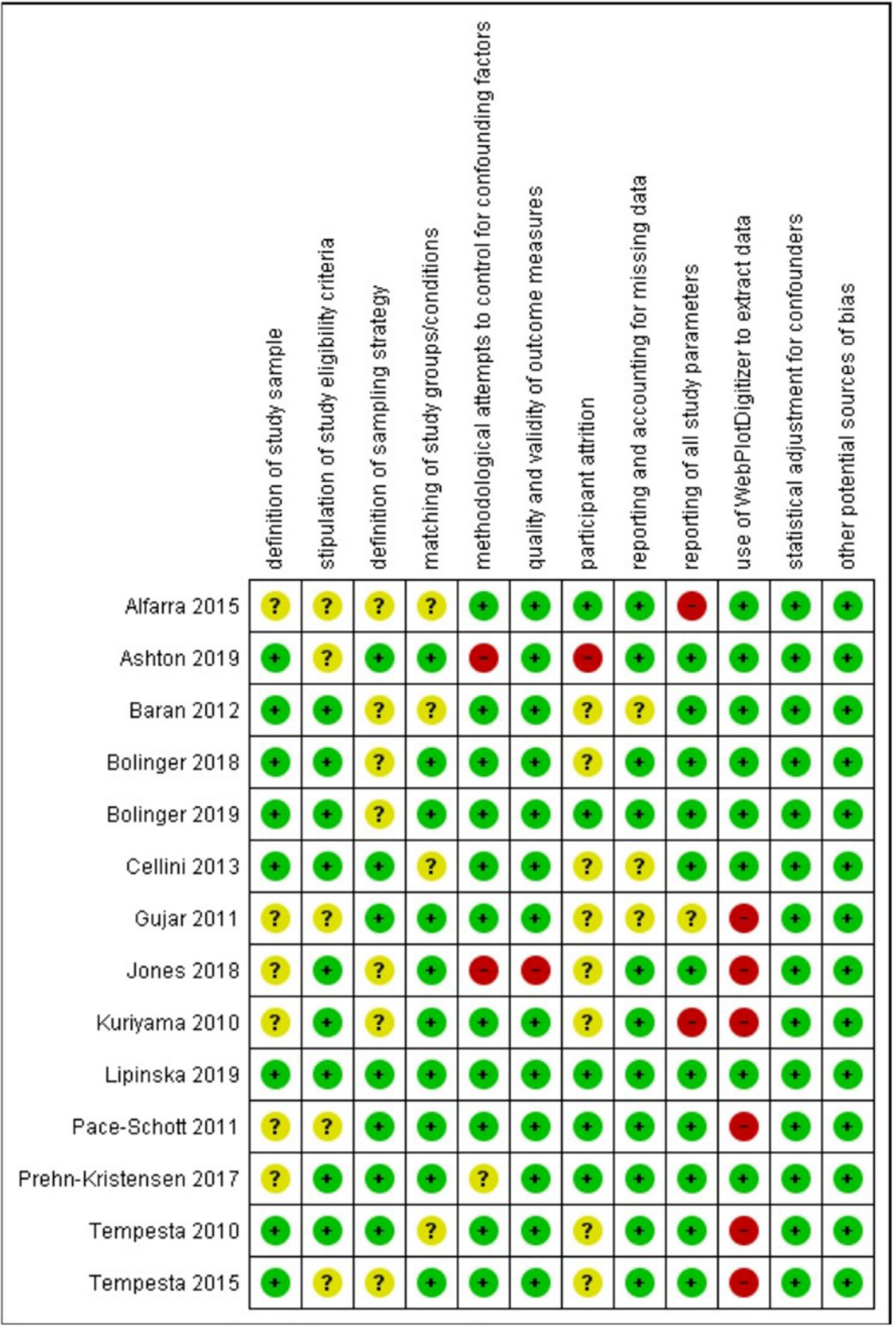
Risk of bias (low, unclear, or high) for each study included in meta-analysis on each rated methodological dimension (*N* = 14).

**Figure 3:**
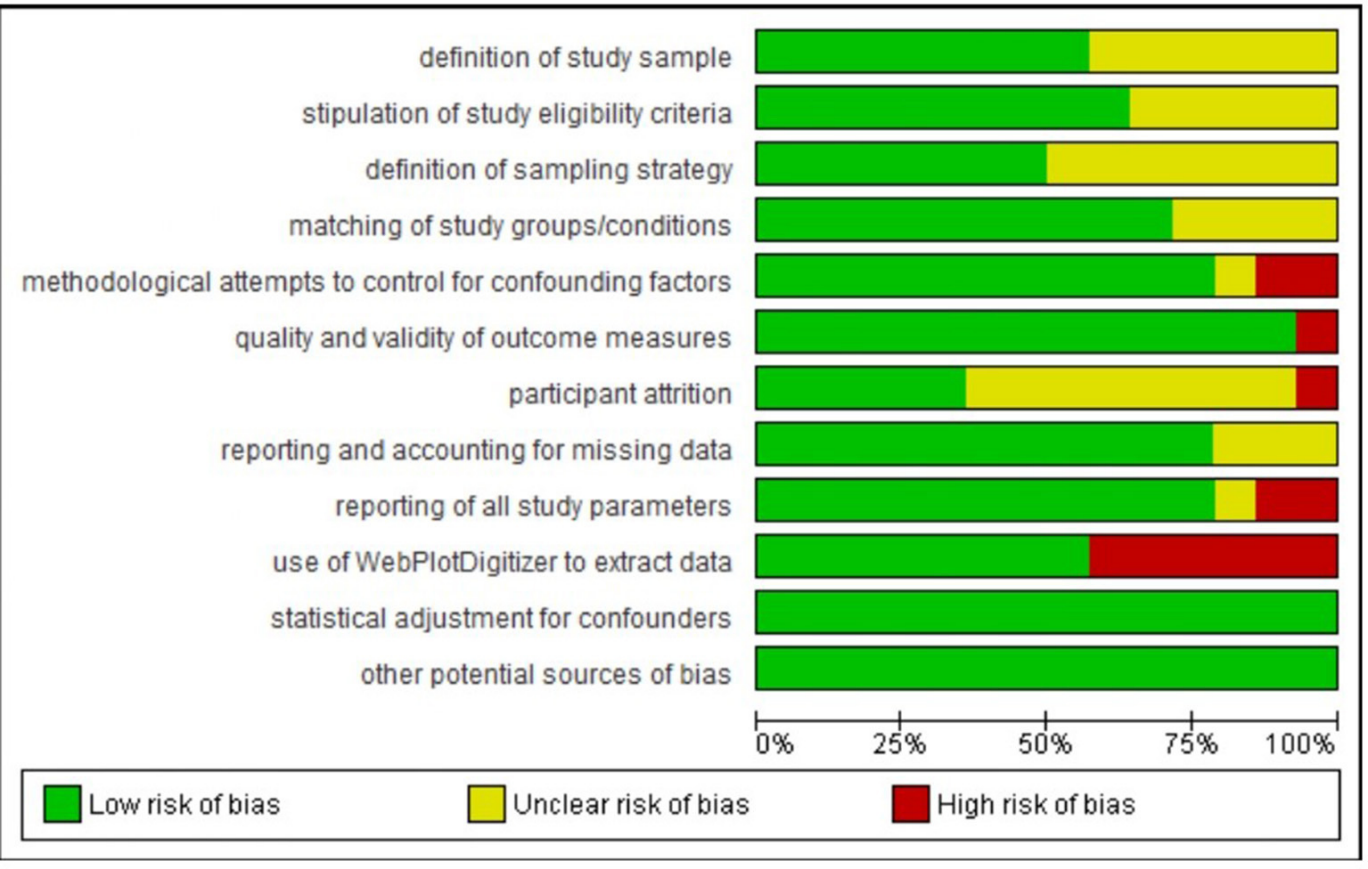
Percentage of studies in the metanalysis rated as being of low, unclear, and high risk of bias on each rated methodological dimension (*N* = 14).

Most of the 24 studies in our review sample were rated as having low or unclear risk of bias on most rated dimensions (see Supplementary Figures 1, 2; Figures 2, 3). As the Figures show, only three studies (Reddy et al., 2014; Hot et al., 2016; Reid et al., 2019) were judged as having an unclear risk of bias across all domains. The descriptions of these studies, which were reported in conference abstracts, provided insufficient information to assess risk of bias. Several studies were judged as having an unclear risk of bias with regard to defining study samples, stipulating eligibility criteria, and reporting on levels of attrition—these studies did not provide adequate information regarding those aspects of their methods. Where studies were judged as having high risk of bias, it was most often a consequence of limited attempts to control for potential confounders, using non-standard measures that were not well described, significant participant attrition, and, in the case of studies included in the meta-analysis, data not being reported in text or tables and therefore needing to be extracted from figures, leading to approximations of *M* and *SD*/*SEM* values. The paragraphs below provide more detail regarding our risk-of-bias ratings.

Regarding *definition of the study sample*, we considered all studies to be of low or unclear risk of bias. Although all studies defined their samples adequately, those rated as unclear on this dimension provided few details regarding recruitment.

Regarding *stipulation of study eligibility criteria and demonstration of how these were enforced*, again we considered all studies to be of low or unclear risk of bias. Studies rated unclear on this dimension usually provided a list of exclusion criteria but gave little information on how participants were screened against these criteria or how their exclusion was ensured.

Regarding *definition of the sampling strategy*, again we considered all studies to be of low or unclear risk of bias. Studies rated unclear on this dimension were those that provided insufficient detail to allow clear judgment on how the sampling proceeded.

Regarding *matching of study groups/conditions on sociodemographic and/or other characteristics*, most studies provided a description of these matching processes and of an assessment of their success. Hence, they were rated as being at low risk of bias on this dimension. The remaining studies were rated as unclear.

Regarding *methodological attempts to control for potential confounding factors*, three studies were rated as being at high risk of bias. Ashton et al. (2019) (a study included in the meta-analysis) did not report including an adaptation night in their study protocol; Jones et al. (2018) (a study included in the meta-analysis) asked participants assigned to their waking comparison conditions not to nap and to limit caffeine between sessions but did not ask participants assigned to their sleep conditions to do similarly; and Kuriyama et al. (2013) did not report considering potential confounders of their outcome. One study included in the meta-analysis was rated as being unclear: Prehn-Kristensen et al. (2017) provided limited information on what instructions participants were given regarding factors such as diet or exercise. All other studies included in the meta-analysis were rated as being at low risk of bias on this dimension, while all other studies were rated as unclear.

Regarding *the quality and validity of outcome measures*, two studies were rated as being at high risk of bias. Jones et al. (2018) (a study included in the meta-analysis) used as stimuli pictures from an apparently unvalidated in-house set alongside IAPS images. In Minkel et al. (2011), it was unclear whether the film clip stimuli were of equivalent valence/arousal over the study period. All studies included in the meta-analysis (other than Jones et al., 2018) were rated as being at low risk of bias on this dimension, while all other studies were rated as being at low or unclear risk.

Regarding *participant attrition*, ratings were particularly difficult to make because most studies in the sample did not report any attrition statistics. (For instance, eight of the 14 studies included in the meta-analysis made no such report.) We decided to rate as unclear those studies that made no report, and as low risk those that made distinct statements indicating that more than 80% of enrolled participants had completed the study protocols. Ashton et al. (2019) (a study included in the meta-analysis) and Lau et al. (2020) were judged as being at high risk of bias on this dimension because it was clear from their study descriptions that fewer than 80% of enrolled participants completed the study.

Regarding *reporting on and accounting for missing data*, all studies were rated as being of low or unclear risk of bias.

Regarding *reporting of all study parameters*, two studies included in the meta-analysis were rated as being at high risk of bias: Alfarra et al. (2015) did not report on key characteristics of their sample, and Kuriyama et al. (2010) did not report fear ratings at baseline. All other studies (bar Gujar et al., 2011, which was rated as unclear) included in the meta-analysis were rated as being at low risk of bias on this dimension.

Regarding *the use of WebPlotDigitizer to extract data*, we had to do so for seven of the 14 studies included in the meta-analysis (Kuriyama et al., 2010; Tempesta et al., 2010, 2015; Gujar et al., 2011; Pace-Schott et al., 2011; Jones et al., 2018) because the data required for meta-analytic calculations were not available in text or tables. Data extracted in this way are an approximation, and so these studies were judged as being at high risk of bias on this dimension.

Regarding *statistical adjustment for confounders*, all studies either used a suitable method to perform such adjustment (and were therefore judged to be at low risk of bias; this includes all studies that formed part of the meta-analysis) or did not describe the methods used (and were therefore judged to have an unclear risk of bias).

Regarding *other potential sources of bias*, all studies either provided information on funding and potential conflicts of interest (and were therefore judged to be at low risk of bias; this includes all studies that formed part of the meta-analysis) or did not report this information (and were therefore judged to have an unclear risk of bias).

### Narrative synthesis

Here, we provide a narrative account of all studies included in the review (i.e., the 10 articles whose study descriptions precluded incorporation of data into the meta-analysis, and the 14 that were included in the meta-analysis).

#### Studies reporting psychophysiological outcomes

Nine studies reported psychophysiological outcomes related to valenced material in comparison to neutral material after a period of sleep or waking. Two of those studies (Cunningham et al., 2014; Lipinska and Thomas, 2019) showed that participants had attenuated HRD and PEP after a period of overnight sleep, in contrast to regular daytime waking activity, although this result was not specific to valenced material.

Five of the nine studies showed that, in response to both valenced and neutral material, there were no post-sleep HRD or SCL differences (Kuriyama et al., 2010, 2013; Hot et al., 2016; Bolinger et al., 2018, 2019). However, three of these studies showed that both HRD and SCL responses to the material were decreased after a period of either regular daytime waking activity or overnight sleep deprivation (Kuriyama et al., 2013; Bolinger et al., 2018, 2019). Bolinger et al. (2018) suggested that this effect was driven by a decrease in reactivity to negative stimuli. The other two studies showed no change in reactivity to all stimuli after a period of either regular daytime waking activity or overnight sleep deprivation (Kuriyama et al., 2010; Hot et al., 2016).

Two studies showed that HRD increased in response to negative stimuli after a period of sleep (either a full night or a 120-min nap) rather than waking (Pace-Schott et al., 2011; Ashton et al., 2019). However, Pace-Schott et al. (2011) indicated that this result was not specific to responses to negative stimuli: The same result was seen in response to neutral stimuli.

Overall, there is no discernible pattern in psychophysiological reactivity to valenced stimuli after a period of sleep rather than an equivalent period of waking.

#### Studies reporting valence ratings

Seventeen studies (incorporating 18 datasets) evaluated self-reported valence ratings in response to emotional and neutral stimuli. Only three of those studies showed less negative reactivity in response to negative rather than neutral stimuli after a period of either overnight sleep or a daytime nap (Gujar et al., 2011; Bolinger et al., 2018; Ashton et al., 2019). However, Ashton et al. (2019) found that this effect was not specific to sleep, as a similar tendency toward more positive ratings of negative stimuli was seen after an equivalent period of waking. In contrast, Schoch et al. (2017) found that participants rated stimuli as less negative after a period of continuous overnight sleep in contrast to continuity-disrupted sleep, but noted that this effect was not specific to valenced material (i.e., it was found with neutral material as well).

Most studies reporting data on valence ratings showed either maintained reactivity in post-sleep responses to emotional stimuli or no effect of either sleep or waking on reactivity. Two studies showed that while a period of overnight sleep maintained responses to negatively valenced material, an equivalent period of regular daytime waking activity resulted in more positive responses to the same stimuli (Jones et al., 2018; Bolinger et al., 2019). Two studies showed the opposite effect: While reactivity to valenced stimuli was maintained after sleep, normal waking (Lau et al., 2020) and sleep deprivation (Tempesta et al., 2015) resulted in more negative responses to negative and positive stimuli respectively. One study showed the same sleep effect, but participants rated neutral stimuli as more negative after a period of sleep deprivation (Tempesta et al., 2010). Six studies showed no effect of sleep (either overnight or nap) or waking (either regular daytime activity or sleep deprivation) on both valenced and neutral material – reactivity was maintained in all conditions (Kuriyama et al., 2010; Minkel et al., 2011; Pace-Schott et al., 2011; Cellini et al., 2016; Prehn-Kristensen et al., 2017; Bolinger et al., 2018).

Only one study showed that after a period of sleep, but not waking, participants were more reactive to valenced stimuli in comparison to neutral stimuli (Wagner et al., 2002). This study showed that (a) after a period of REM-rich sleep, participants showed enhanced reactivity to negative stimuli, whereas (b) after a period of sleep rich in slow waves, their responses were enhanced for positive stimuli.

In summary, most studies showed either maintained reactivity to valenced material after a period of sleep, rather than waking, or no effect of sleep or waking on valenced or neutral material.

#### Studies reporting arousal ratings

Ten studies (incorporating 11 datasets) evaluated self-reported arousal ratings in response to emotional and neutral stimuli. Of these studies, two reported decreased arousal after a period of overnight sleep (Bolinger et al., 2018, 2019). However, in both cases this decrease was not specific to valenced material (i.e., it was also observed in response to neutral material), and in one case it was not specific to sleep (i.e., it was also observed after a period of regular daytime waking activity).

One study showed that a full night of sleep maintained arousal in response to all stimuli, but that total night-time sleep deprivation resulted in increased self-reported arousal to these stimuli (Tempesta et al., 2010). Four studies (incorporating five datasets) showed no effect of sleep (either overnight or nap) or waking (either regular daytime activity or sleep deprivation) on arousal responses to both emotional and neutral material (Tempesta et al., 2015; Cellini et al., 2016; Jones et al., 2018; Ashton et al., 2019).

Two studies showed increased arousal after a period of sleep. Baran et al. (2012) reported that this effect was specific to negatively valenced stimuli and that it was more pronounced after a full night of sleep in contrast with a full day of waking activity. However, Schoch et al. (2017), making an identical sleep-wake comparison, showed no effect of valence or of condition.

In summary, there is little consensus regarding attenuation, maintenance, or enhancement of arousal in response to valenced material after a period of sleep. Most studies in this group do, however, report no effect of valence and condition on self-reported arousal ratings.

### Meta-analysis

#### Studies reporting psychophysiological outcomes

Five studies (Kuriyama et al., 2010; Pace-Schott et al., 2011; Bolinger et al., 2018; Ashton et al., 2019; Lipinska and Thomas, 2019; total *N* = 96) measured psychophysiological outcomes. Overall, this group of studies indicated that neither sleep nor equivalent periods of wakefulness had a statistically significant effect on emotional reactivity to either negatively valenced or neutral stimuli.

**Figure 4:**
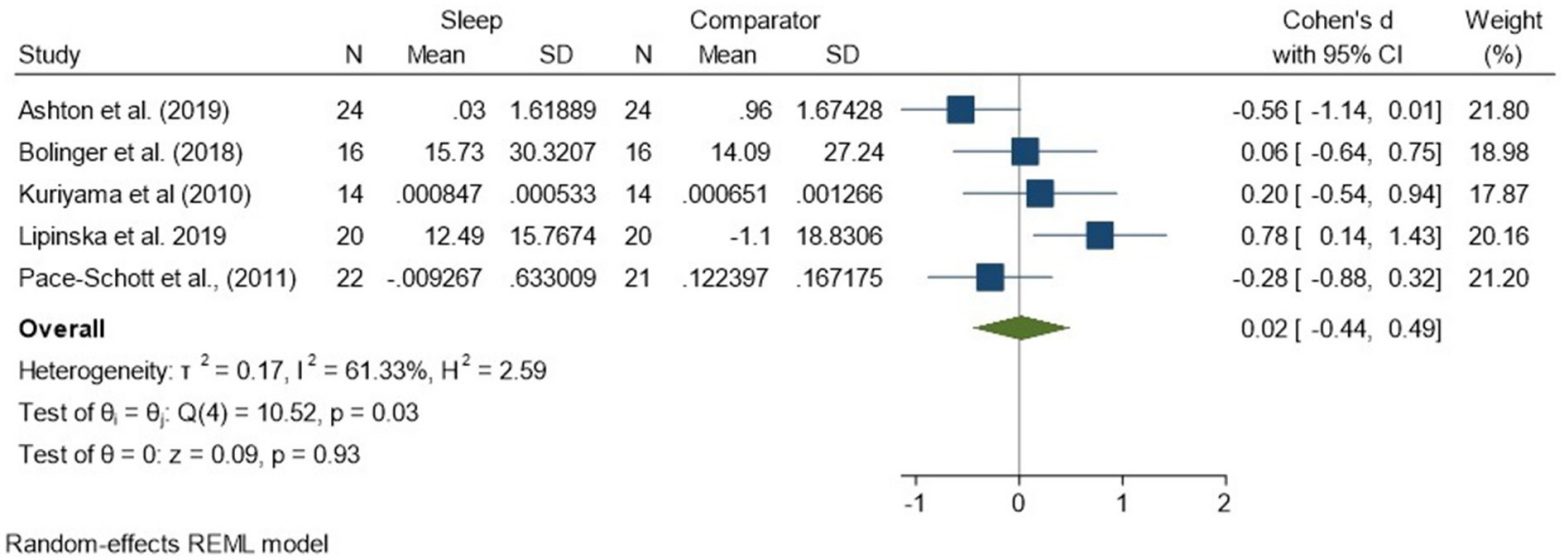
Meta-analysis I: Physiological outcomes in response to negative stimuli – changes across a period of sleep compared to across a period of waking (*K* = 5, *N* = 155).

Regarding analyses of changes in reactivity to negatively valenced material over a period of sleep compared to changes over an equivalent comparison period, there was a standardized effect size of 0.02 (95% CI −0.44, 0.49) and no statistically significant difference (*p* = 0.93, *I*^2^ = 61%; see Figure 4). Because three of the five studies used the same outcome measure (HRD), we examined the collective results of these three studies (Pace-Schott et al., 2011; Bolinger et al., 2018; Ashton et al., 2019). These results were more consistent, with a small-to-moderate effect size of −0.30 (95% CI −0.66, 0.06). However, they still failed to meet the threshold for statistical significance (*p* = 0.10, *I*^2^ = 0%), perhaps due to the small sample size (*n* = 62; see Supplementary Figure 3).

**Figure 5:**
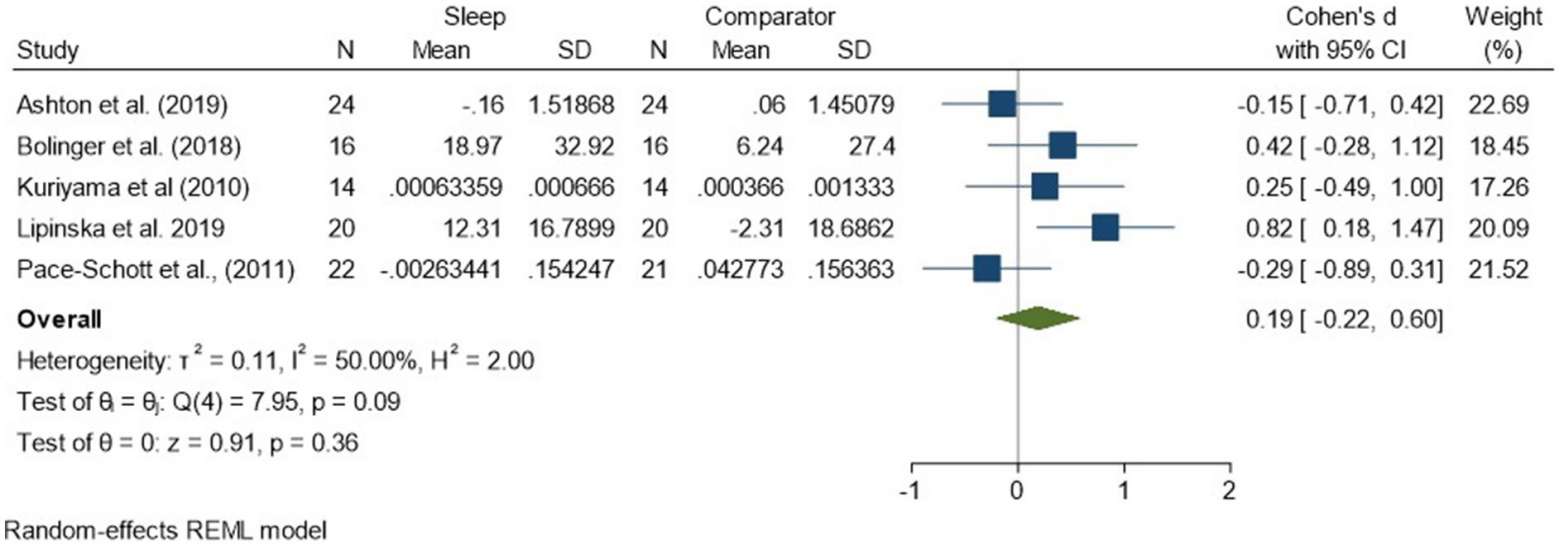
Meta-analysis II: Physiological outcomes in response to neutral stimuli – changes across a period of sleep compared to across a period of waking (*K* = 5, *N* = 155).

Regarding analyses of changes in reactivity to neutral material over a period of sleep compared to changes over an equivalent comparison period, there was a standardized effect size of 0.19 (95% CI −0.22, 0.60) and no statistically significant difference (*p* = 0.36, *I*^2^ = 50%; see Figure 5). Analysis of the three studies reporting HRD measures only revealed a very small effect size of −0.05 (95% CI −0.42, 0.33) that was not statistically significant (*p* = 0.80, *I*^2^ = 9%; see Supplementary Figure 4).

#### Studies reporting valence ratings

The studies described in this subsection evaluated changes in reactivity (as indexed by self-reported valence ratings) to negative, neutral, or positive material over a period of sleep compared to changes over an equivalent comparison period. The three separate analyses (one for each stimulus type) all detected small-to-moderate effects sizes that were not statistically significant.

**Figure 6:**
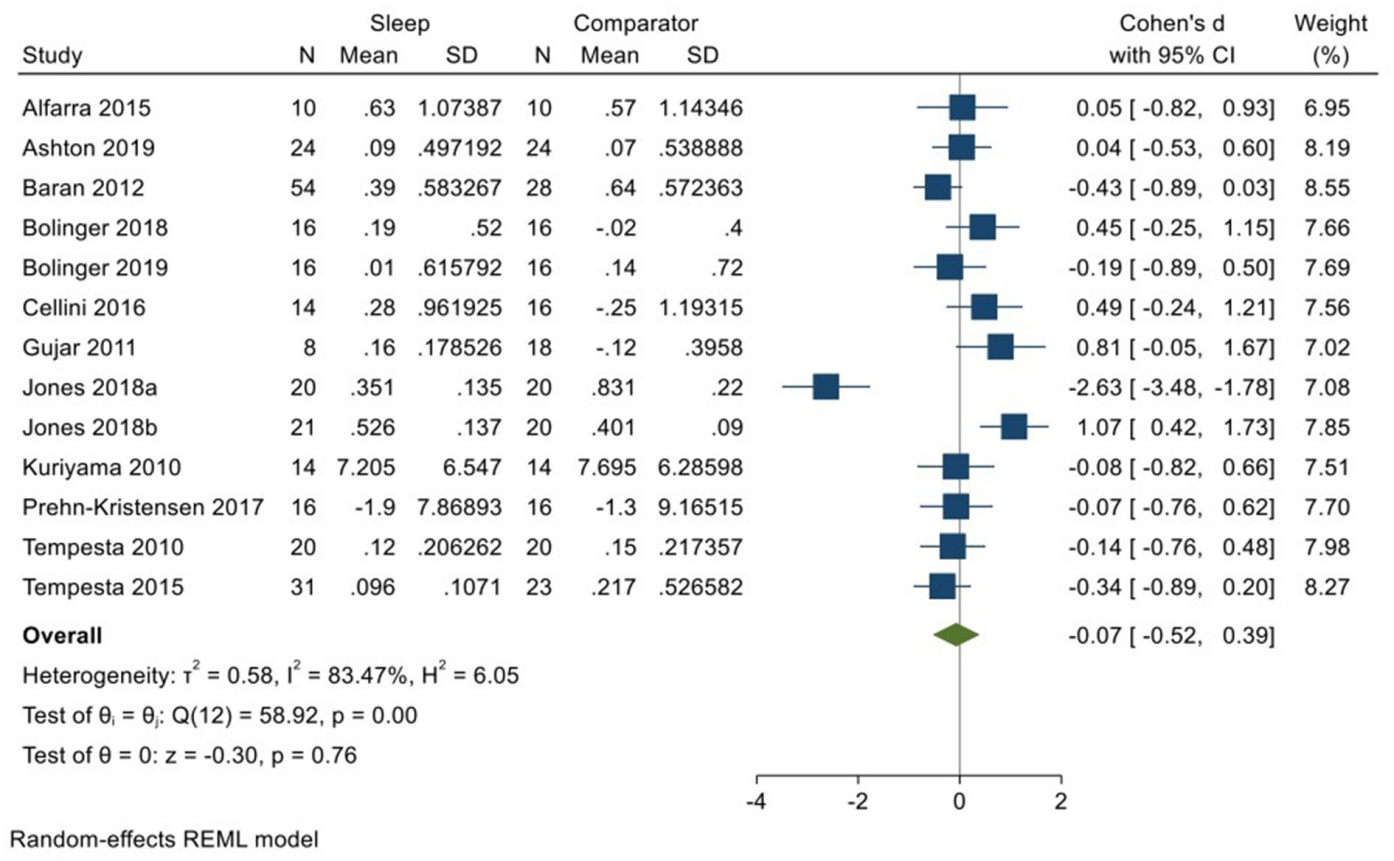
Meta-analysis III: Outcomes for self-reported valence ratings in response to negative stimuli – changes across a period of sleep compared to across a period of waking (*K* = 13, *N* = 463).

Twelve studies incorporating 13 datasets (Kuriyama et al., 2010; Tempesta et al., 2010, 2015; Gujar et al., 2011; Baran et al., 2012; Alfarra et al., 2015; Cellini et al., 2016; Prehn-Kristensen et al., 2017; Bolinger et al., 2018, 2019; Jones et al., 2018; Ashton et al., 2019; total *N* = 297) evaluated changes in reactivity to negatively valenced material over a period of sleep compared to changes over an equivalent comparison period. Analysis of those data detected a very small effect size of −0.07 (95% CI −0.52, 0.39) that was not statistically significant (*p* = 0.76; *I*^2^ = 84%; see Figure 6).

**Figure 7:**
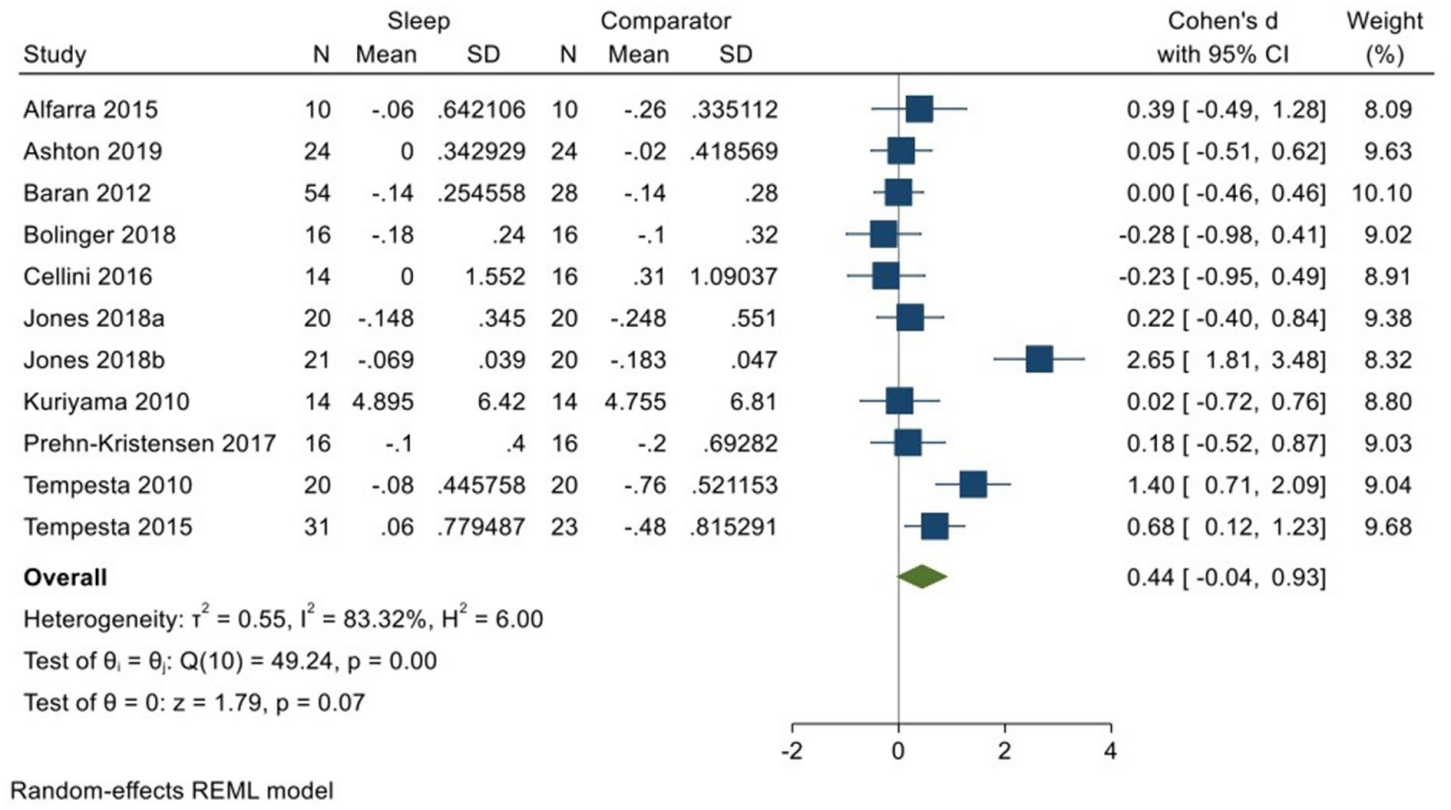
Meta-analysis IV: Outcomes for self-reported valence in response to neutral stimuli – changes across a period of sleep compared to across a period of waking (*K* = 11, *N* = 405).

Ten studies incorporating 11 datasets (Kuriyama et al., 2010; Tempesta et al., 2010, 2015; Baran et al., 2012; Alfarra et al., 2015; Cellini et al., 2016; Prehn-Kristensen et al., 2017; Bolinger et al., 2018; Jones et al., 2018; Ashton et al., 2019; total *N* = 240) evaluated changes in reactivity to neutral material over a period of sleep compared to changes over an equivalent comparison period. Analysis of those data detected a moderate effect size of 0.44 (95% CI −0.04, 0.93) that was not statistically significant (*p* = 0.07, *I*^2^ = 83%; see Figure 7).

**Figure 8:**
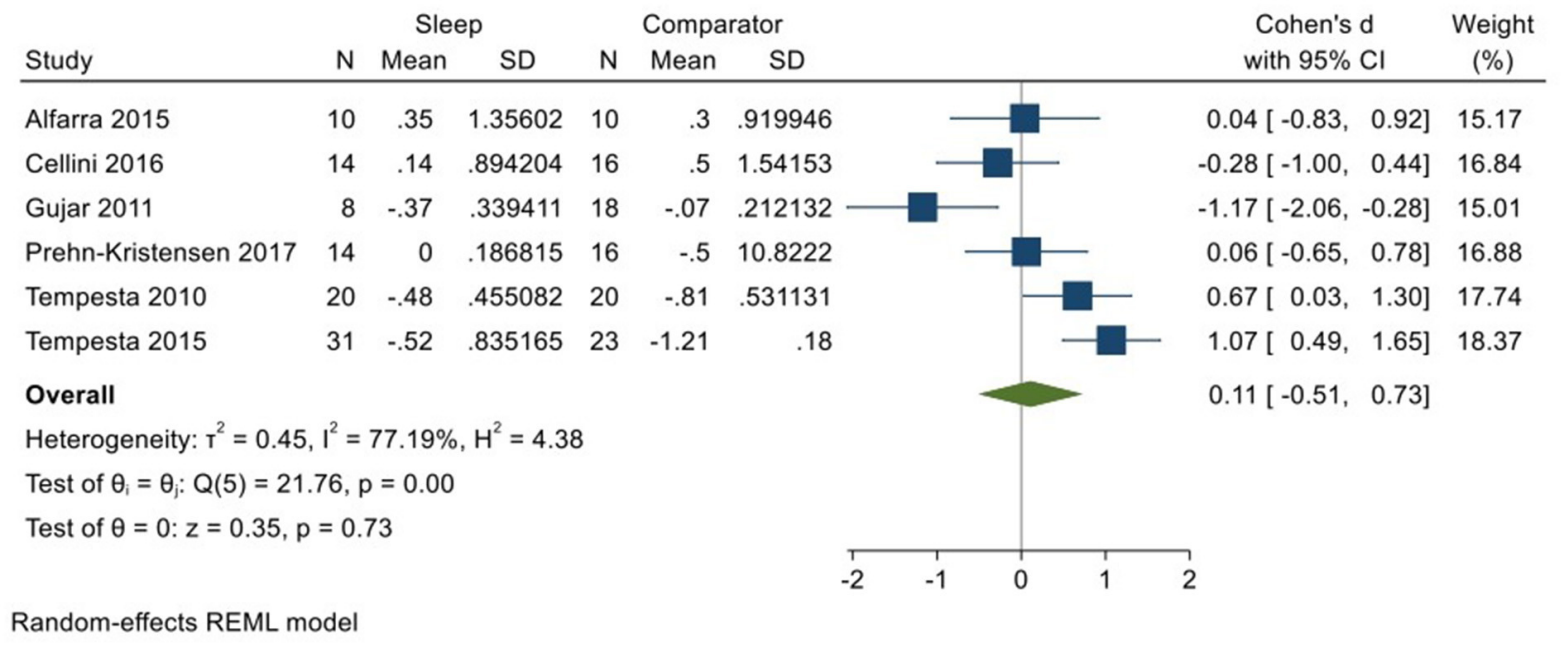
Meta-analysis V: Outcomes for self-reported valence ratings in response to positive stimuli – changes across a period of sleep compared to across a period of waking (*K* = 6, *N* = 176).

Six studies (Tempesta et al., 2010, 2015; Gujar et al., 2011; Alfarra et al., 2015; Cellini et al., 2016; Prehn-Kristensen et al., 2017; total *N* = 97) evaluated changes in reactivity to positively valenced material over a period of sleep compared to changes over an equivalent comparison period. Analyses of those data detected a small effect size of 0.11 (95% CI −0.51, 0.73) that was not statistically significant (*p* = 0.73, *I*^2^ = 77%; see Figure 8).

#### Studies reporting arousal ratings

The studies described in this sub-section evaluated changes (as indexed by self-reported arousal ratings) in reactivity to negative, neutral, or positive material over a period of sleep compared to changes over an equivalent comparison period. Only analyses of data comprising negative material detected a statistically significant effect; analyses of positive and neutral data detected small and non-significant effects.

**Figure 9:**
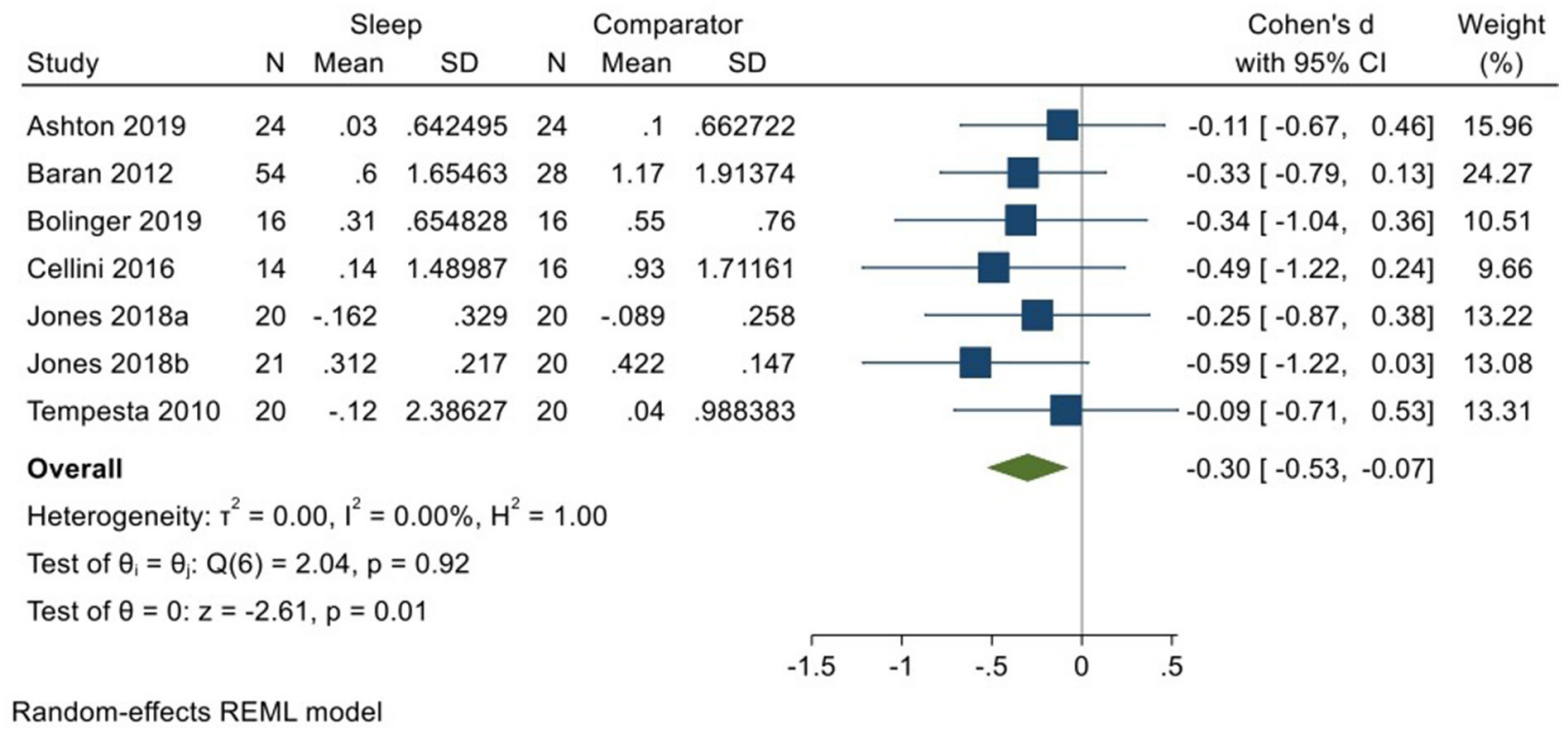
Meta-analysis VII: Outcomes for self-reported arousal ratings in response to negative stimuli – changes across a period of sleep compared to across a period of waking (*K* = 7, *N* = 313).

Six studies incorporating 7 datasets (Kuriyama et al., 2010; Tempesta et al., 2010; Baran et al., 2012; Cellini et al., 2016; Jones et al., 2018; Ashton et al., 2019; Bolinger et al., 2019; total *N* = 169) evaluated changes in reactivity to negatively valenced material over a period of sleep compared to changes over an equivalent comparison period. Analysis of those data detected a modest effect size of −0.30 (95% CI −0.53, −0.07) that was statistically significant (*p* = 0.01, *I*^2^ = 0%; see Figure 9).

**Figure 10:**
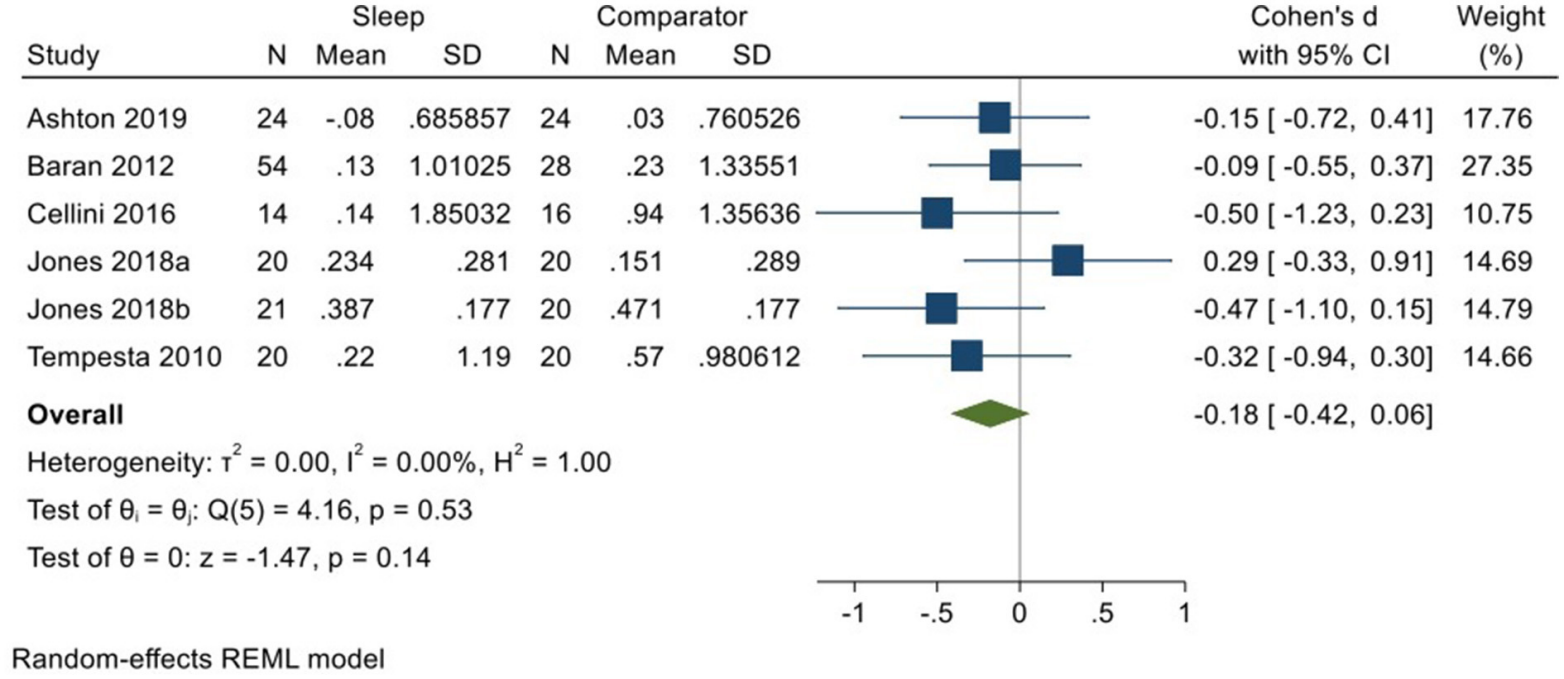
Meta-analysis VIII: Outcomes for self-reported arousal ratings in response to neutral stimuli – changes across a period of sleep compared to across a period of waking (*K* = 6, *N* = 281).

Five studies incorporating 6 datasets (Tempesta et al., 2010; Baran et al., 2012; Cellini et al., 2016; Jones et al., 2018; Ashton et al., 2019; total *N* = 153) evaluated changes in reactivity to neutral material over a period of sleep compared to changes over an equivalent comparison period. Analysis of those data detected a small effect size of −0.18 (95% CI −0.42, 0.06) that was not statistically significant (*p* = 0.14, *I*^2^ = 0%; see Figure 10).

**Figure 11:**
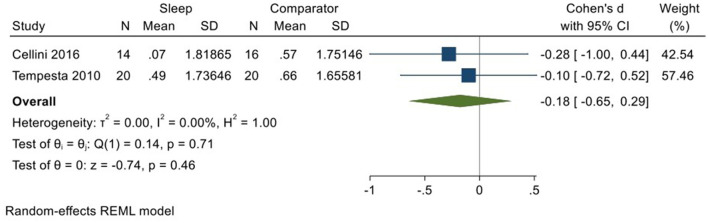
Meta-analysis IX: Outcomes for self-reported arousal ratings in response to positive stimuli – changes across a period of sleep compared to across a period of waking (*K* = 2, *N* = 70).

Two studies (Tempesta et al., 2010; Cellini et al., 2016; total *N* = 34) evaluated changes in reactivity to positively valenced material over a period of sleep compared to changes over an equivalent comparison period. Analysis of those data detected a small effect size of −0.18 (95% CI −0.65, 0.29) that was not statistically significant (*p* = 0.46, *I*^2^ = 0%; see Figure 11).

**Figure 12:**
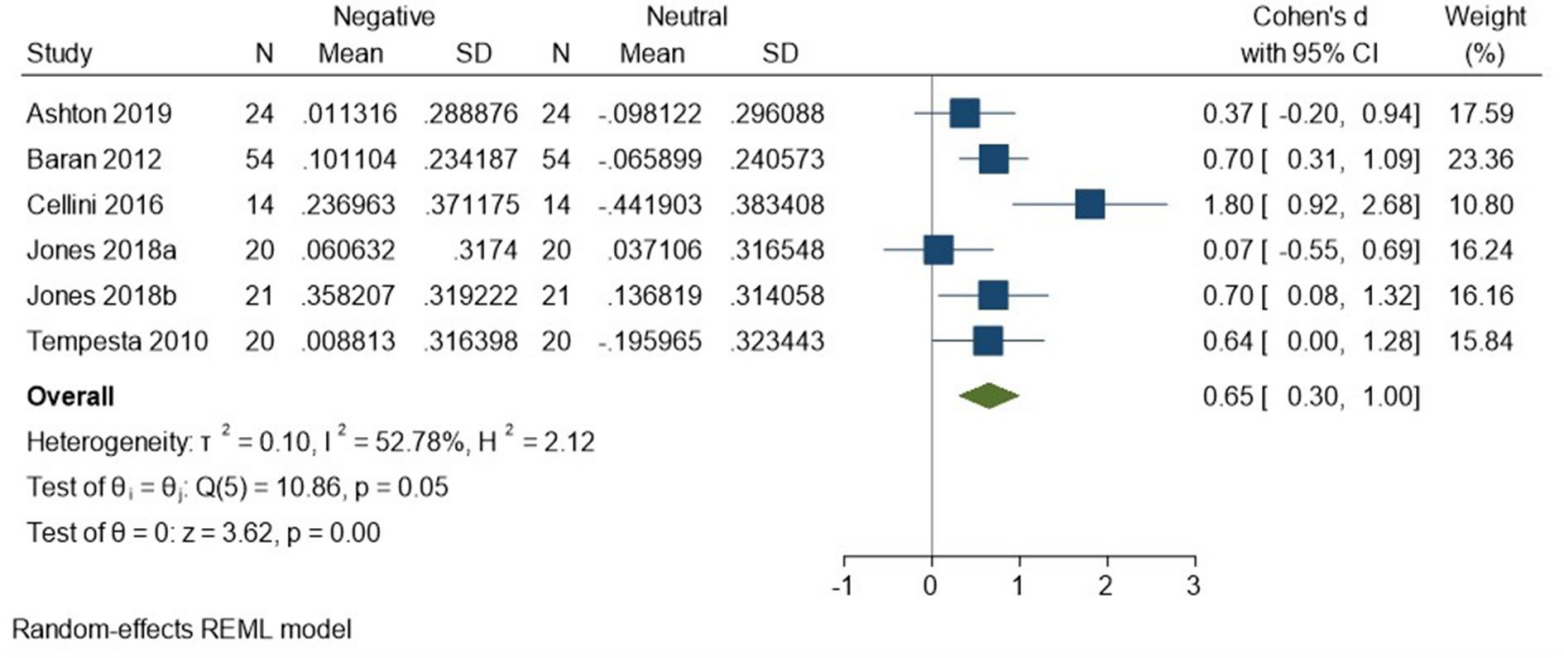
Meta-analysis X: a 2 (condition: sleep, waking) × 2 (valence: negative, neutral) comparison of self-reported arousal ratings (*K* = 6, *N* = 281).

There appears to be preferential regulation during sleep of self-reported arousal in response to negatively valenced material. We compared the SMC from the negative condition in the sleep-vs.-waking analysis to the SMC from the neutral condition in the sleep-vs.-waking analysis. Five studies comprising six datasets (Tempesta et al., 2010; Baran et al., 2012; Cellini et al., 2016; Jones et al., 2018; Ashton et al., 2019; total *N* = 153) contributed data to this analysis, which detected a moderate effect size of 0.65 (95% CI 0.30, 1.00) that was statistically significant (*p* < 0.001, *I*^2^ = 53%; see Figure 12).

#### Subgroup analyses

We examined whether age, gender, data extraction method (numerical or graphic), type of waking control (sleep deprivation vs. ordinary daytime waking), sleep duration (whole night vs. nap), kind of emotional stimuli (IAPS vs. other) and whether participants obtained REM sleep (either typical whole-night REM percentage or reported REM sleep in a nap paradigm) influenced self-reported valence and arousal outcomes.

Regarding psychophysiological outcomes, there was too little variation in these moderator variables between studies for us to conduct sub-group analyses. For example, Pace-Schott et al. (2011) was the only study in this group that required data extraction from figures. In four of the five studies, the research design featured a daytime waking control period; the only exception was Kuriyama et al. (2010), which used a period of sleep deprivation. Similarly, four of the five study designs featured a whole-night sleep condition; the only exception was Pace-Schott et al. (2011), which used a nap condition. All five studies used IAPS stimuli. Finally, no study except Bolinger et al. (2018) presented REM data.

Regarding self-reported valence and arousal outcomes, analyses detected statistically significant subgroup effects for valence outcomes only. Three moderators had an influence on valence ratings: the kind of comparison condition used (daytime waking vs. sleep deprivation), the duration of sleep (whole night vs. nap), and whether participants experienced REM sleep or not (see Supplementary Figures 5–22 for details regarding all subgroup analyses).

Regarding the effect of the kind of waking comparison condition, the sub-group analysis showed that in response to positive stimuli (but not in response to negative or neutral stimuli, *p* = 0.78 and *p* = 0.64, respectively; see Supplementary Figures 5, 6), those who were sleep deprived tended to have significantly larger changes in reactivity than those who experienced a full night of sleep (*p* = 0.01; see Supplementary Figure 7). The study data showed that these enhanced responses were characterized by a more negative response to positive stimuli after sleep deprivation. However, when the comparison condition was daytime waking, participants tended to have increased reactivity, albeit somewhat unreliably, to positive stimuli after a period of sleep rather than waking.

Subgroup analyses examining the effect of sleep duration revealed that participants tended to have attenuated reactivity in response to negative stimuli after a nap, rather than an equivalent period of waking, and a slight but inconsistent increase in reactivity after a whole night of sleep (*p* = 0.04; see Supplementary Figure 8). However, the analyses showed an opposite effect for ratings in response to positive stimuli: Whereas participants responded more strongly to positive stimuli after a nap compared to a period of waking, after a whole night of sleep compared to a period of waking, they tended to have attenuated responses to these stimuli (*p* = 0.02; see Supplementary Figure 10). Notably, however, the differences observed in this analysis may be due to the effects of the comparator condition, given that all the studies using a nap paradigm had daytime waking as a control whereas almost all the studies using the whole night method used sleep deprivation as the comparison. There was no significant subgroup effect for valence ratings in response to neutral stimuli.

Finally, participants who obtained REM sleep, compared to those who did not spend any significant time in this sleep stage, did not react significantly differently to negative material in a sleep-vs.-waking analysis (*p* = 0.76; see Supplementary Figure 14). However, a similar analysis of data for reactivity to neutral material detected a significant subgroup effect (*p* = 0.04; see Supplementary Figure 15). This finding suggests that participants who did not obtain a substantial amount of REM sleep (i.e., spent a larger proportion of their sleep time in non-REM stages) showed attenuated reactivity to neutral stimuli after a period of sleep but not after a period of ordinary daytime wakefulness or sleep deprivation. No such effect was observed for participants who obtained a substantial amount of REM sleep. Regarding reactivity in response to positive material, there was a significant difference between those that experienced REM sleep and those that were less likely to experience this stage of sleep, *p* = 0.01 (see Supplementary Figure 16). However, this effect overlaps almost entirely with the difference seen between studies that used a sleep deprivation versus daytime waking control condition, and as a result should not be interpreted as a REM-specific result.

### Publication bias

Only the negative and neutral valence comparisons had a sufficient number of included studies to explore evidence for possible publication bias. The funnel plots (see Supplementary Figures 23, 24) suggested that although there were some outliers with large effect sizes, there was no systematic evidence of such bias. Results from Egger's test also suggested no evidence for small study effects in both the negative (*p* = 0.79) and neutral (*p* = 0.21) comparisons.

## Discussion

Previously published studies investigating whether sleep regulates spontaneous emotional regulation have produced contradictory results: it is unclear whether a period of sleep experienced subsequent to encounters with emotionally valenced stimuli will, upon further exposure to those stimuli, attenuate, enhance, or simply maintain reactivity to them. Hence, we set out to systematically review and meta-analyze published work investigating whether sleep, without cognitive modulation, acts to regulate emotion. We reviewed studies that reported emotional reactivity for negatively and/or positively valenced material compared to neutral material over any period of sleep (whole night or nap) compared to a matched period of waking or sleep deprivation (i.e., wakefulness during either the day or the night). Given the broader literature indicating that sleep restriction impacts mood negatively, we hypothesized that our review would show that sleep preferentially down-regulates or ameliorates reactivity to emotional stimuli over neutral stimuli, and that this down-regulation or amelioration is greater than what is observed over equivalent periods of waking.

Broadly speaking, our results did not confirm this prediction. Sleep (or, indeed, equivalent periods of wakefulness) did not have a statistically significant effect on physiological measures of emotional reactivity to either negatively valenced or neutral stimuli. However, sleep did have a statistically significant effect on self-report measures of emotional reactivity, albeit not always in the predicted direction. Specifically, arousal ratings in response to negatively valenced stimuli (but not positively valenced or neutral stimuli) were significantly higher after a period of sleep but not after an equivalent period of waking.

Sub-group analyses of data regarding self-reported valence and arousal outcomes indicated that several important moderators influenced differences in emotional reactivity between sleep and waking conditions. First, participants who were sleep deprived in comparison to those who slept a full night rated positive stimuli more negatively, but no such between-condition difference was significant when the comparison condition was a period of daytime wakefulness. Second, participants in nap conditions in comparison to those who experienced an equivalent period of waking rated negative pictures less negatively. Participants showed a slight but inconsistent increase in reactivity after a whole night of sleep, in comparison with waking conditions. Third, participants who did not spend a significant amount of time in REM sleep tended to provide attenuated valence ratings in response to neutral stimuli after a period of sleep, rather than waking. This effect was not observed in those who did achieve REM sleep.

### Studies reporting psychophysiological outcomes

Our finding that sleep did not have a statistically significant effect on changes in psychophysiological reactivity to negatively valenced or neutral stimuli is consistent with results reported previously in this literature. Several single empirical studies have shown that HRD or SCL responses to valenced and neutral material were not significantly different before and after a period of sleep (Kuriyama et al., 2010, 2013; Hot et al., 2016; Bolinger et al., 2018, 2019). However, three of these studies showed that HRD in response to such material decreased over a period of waking (Kuriyama et al., 2013; Bolinger et al., 2018, 2019). Furthermore, Bolinger et al. (2018) showed that this effect was driven by a decrease in reactivity to negative stimuli in particular.

Our meta-analysis detected a non-significant trend toward increased HRD in response to negatively valenced stimuli after a period of sleep relative to an equivalent period of waking. Some authors have found significantly enhanced HRD in response to negatively valenced material after sleep (Pace-Schott et al., 2011; Ashton et al., 2019), although the latter noted that this pattern of data was also observed for neutral stimuli.

In summary, the cumulative results from this group of studies suggest that psychophysiological measures of changes in emotional reactivity do not consistently detect any significant effects of sleep, and that when they do detect these effects, they are not specific to valenced materials. Although one psychophysiological measure (HRD) does appear relatively more sensitive to sleep-associated changes in emotional reactivity, effects are not strong or consistent and it remains unclear whether they are specific to valenced material.

### Studies reporting valence and arousal rating outcomes

Our finding of the trend toward post-sleep enhanced HRD in response to negative material is mirrored by our findings from the data regarding self-reported arousal ratings. Our analyses indicated that, for negative material to a significantly greater extent than neutral material, these ratings were also higher after a period of sleep compared to a period of waking. Overall, these results suggest that self-reported arousal is a sensitive measure of changes in emotional reactivity across periods of sleep.

This finding is consistent with some results reported previously in the literature. For example, Kuriyama et al. (2010) and Alfarra et al. (2015) found that emotionally valenced pictures were rated as having less emotional charge after a period of sleep deprivation.

This common trend across the psychophysiological and self-report data (i.e., distinct post-sleep increases in arousal responses to negatively valenced material) may reflect a type of environmental adaptation. In healthy adults, enhanced arousal after sleep may contribute to an emotional “next-day readiness” that allows the person an increased sensitivity to emotional stimuli in the environment. Future studies could examine sleep-dependent emotional processing in the context of the cortisol awakening response, which is a biological measure of next-day readiness (Xiong et al., 2021). It should be noted that none of the studies included in the meta-analysis investigated the impact of cortisol levels on emotion reactivity.

However, post-sleep enhancement of self-reported arousal to negative material is not found commonly in this literature. In fact, the opposite result is reported more frequently. For example, Gujar et al. (2011) showed that, in participants who had taken a 90-min nap and obtained REM sleep, post-vs. pre-sleep ratings of fearful expressions were lower (although ratings of angry expressions had not changed and ratings of happy expressions were higher). A different pattern of reactivity was observed in participants who had an afternoon of normal waking activity: They showed amplified reactivity to angry and fearful faces originally viewed earlier in the day.

The current finding of no significant effect of sleep on self-reported valence ratings for negative, positive, and neutral material is, unsurprisingly, consistent with the findings reported by most previous studies. Those studies tend to show either maintained reactivity to valenced material after a period of sleep compared to a period of waking, or no effect of either sleep or waking on both valenced and neutral material (e.g., Tempesta et al., 2015; Cellini et al., 2016; Prehn-Kristensen et al., 2017).

Our sub-group analyses of self-report outcomes suggested that the kind of waking comparison condition (daytime waking or nighttime waking, i.e., sleep deprivation), the duration of sleep (nap or whole night), and the presence/absence of REM sleep all had an impact on self-reported valence ratings.

Regarding the use of daytime waking or sleep deprivation as a comparison to the sleep condition, ideally we would have sought to examine the effects of each kind of comparison condition separately – sleep loss may have different effects on emotion regulation than the passage of ordinary wakefulness (Baran et al., 2012; Motomura et al., 2013). However, our sample did not include enough studies featuring each different comparison condition to examine these effects independently. Nonetheless, sub-group analyses revealed that the comparison between sleep and daytime waking differed from that between sleep and sleep deprivation with respect to self-reported valence ratings in response to positive stimuli. Whereas participants who were sleep deprived tended to respond significantly more negatively to positive stimuli in comparison to their sleep-condition counterparts, those who experienced daytime wakefulness tended to show a more neutral response to these stimuli than those who slept, who showed higher positive ratings. These findings are consistent with several studies indicating that there are specific decreases in positive emotion after sleep deprivation (McMakin et al., 2016; Finan et al., 2017). Furthermore, the finding that individuals may experience increased reactivity to positive stimuli after sleep in comparison with waking is consistent with our main finding of increased arousal (self-report and, to a lesser degree, psychophysiological) after sleep rather than waking.

Regarding sleep duration, we found that having a nap or experiencing a full night of sleep had differing effects on self-reported valence ratings in response to negative stimuli. Whereas a nap (in comparison with an equivalent period of daytime waking) tended to decrease valence ratings in response to these stimuli, a full night of sleep (in comparison to either daytime walking or night-time sleep deprivation) slightly increased valence ratings. These results suggest that responses to previously encountered negative stimuli are modulated differently by a daytime nap and a full night of sleep. One possible mechanism underlying this effect may be the differing proportions of NREM and REM sleep or spindle quality during napping and whole-night sleep (van Schalkwijk et al., 2019).

Finally, sub-group analyses indicated that the presence or absence of REM sleep moderated changes in emotional reactivity for neutral material. Participants who did not obtain substantial REM sleep during either a whole night of sleep or a daytime nap had attenuated reactivity to neutral stimuli, whereas the same effect was not found in those who either (a) did obtain substantial REM sleep, or (b) were exposed to sleep deprivation or ordinary wakefulness. This result may point to differential effects of REM and NREM sleep stages on sleep-dependent emotional processing, with NREM sleep possibly more responsible for processing neutral information and down-regulating responses to such stimuli.

### Limitations and directions for future research

Perhaps the most consequential limitation of the foundational literature, certainly in terms of its influence on our review, is the vast cross-study differences in methodology. These methodological variations include the timing and duration of the sleep condition, the type of waking control used, the kind of emotional stimulus presented, the primary outcome measured, and whether participants obtained REM sleep (a stage previously observed to be central to the emotional regulatory benefits of sleep; Palagini et al., 2013; Altena et al., 2016). The consequence of these variations is heterogeneity among reported results and limited power to draw conclusions from a meta-analysis.

A second potential limitation is that the relatively small sample sizes within each of our quantitative analyses meant we could not fully explore the bounds of our meta-analysis. For instance, when considering data from studies reporting psychophysiological outcomes, we could not conduct subgroup analyses investigating potential moderators of the effect of sleep on emotional reactivity. One potential moderator here is whether participants in the comparison condition experienced sleep deprivation (i.e., nighttime waking) or ordinary daytime waking, but because all the psychophysiological studies included in our sample used a waking control group we could not investigate further. Other potential moderators are sleep duration and whether participants in the sleep condition obtained REM sleep, but again only one psychophysiological study in our sample used a nap rather than a whole night condition and only one presented REM-specific data. Given this lack of sub-group analyses, we cannot comment fully on the question of under which specific experimental conditions particular effects might be observed.

A third potential limitation is that, in most studies included in our review sample (and, notably, in every psychophysiological study), participants viewed many IAPS pictures. Hence, after a certain point they may have become desensitized to the valenced material and might have begun to evince similar reactions to those images as to neutral images. This consequence of desensitization might explain why the analyses did not consistently detect differences in emotional reactivity to valenced vs. neutral material.

A fourth potential limitation is that not all studies in our sample controlled for time-of-day effects. These influence performance on a variety of cognitive tasks, including emotion regulation (Van Dongen and Dinges, 2003; Schmidt et al., 2007). Additionally, diurnal variations in emotional reactivity are associated with similar time-based changes in autonomic and sympathetic nervous system functioning, with some studies reporting increased reactivity to negative stimuli at “off peak” times of day (Hot et al., 2005; Tucker et al., 2012). Overall, there is a need for more research evaluating interactions between physiological states and emotion regulation to help discern the chronopsychophysiology of emotional processing.

Similarly, not all studies in our sample controlled for sleep history, sleepiness, and vigilance. Of the 14 studies included in the meta-analysis, only half reported methodological attempts to control for these potential confounders. Even the studies that did report implementing such controls took only between-group measures of sleepiness, psychomotor vigilance, and current subjective mood, and did so inconsistently. Furthermore, although most studies asked participants to maintain a regular sleep schedule prior to the starting the experimental procedures, not all measured whether this was achieved. Therefore, it is possible that circadian factors other than sleep had an influence on emotional reactivity. Future studies should strictly control for these factors.

## Summary and conclusion

Overall, our systematic review and meta-analysis indicates that sleep (or, indeed, equivalent periods of wakefulness) does not have a statistically significant effect on psychophysiological measures of emotional reactivity, to either valenced or neutral stimuli. However, self-reported arousal ratings in response to negatively valenced stimuli (but not positively valenced or neutral stimuli) were significantly higher after a period of sleep but not after an equivalent period of waking. Sub-group analyses suggested that the kind of waking comparison condition (regular daytime waking or nighttime sleep deprivation), sleep duration (nap or whole night), and the presence/absence of REM sleep all had an impact on self-reported valence ratings.

Taken together, these results suggest that sleep may have a larger effect on subjective emotional experience than on objectively measured physiological experiences of emotion. In other words, sleep might impact more on responses that are subject to cognitive control than on those that are generated automatically. A caution here is that this speculation is influenced by the fact that a relatively small number of studies in this literature have taken psychophysiological measures.

More consistency in study methodology is needed before the field can gain a better understanding of how sleep impacts reactivity to emotionally valenced information. Future research studies should endeavor to collect both psychophysiological (with an emphasis on HRD) and self-report measures, to report and collect REM sleep parameters, to report all different valence- and condition-specific data independently rather than in aggregate form and control for circadian and time-of-day effects.

A number of other important questions remain unanswered in the field. First, underlying mechanisms explaining sleep-dependent modulation of emotional reactivity remain poorly understood. These mechanisms may be numerous and anatomically widespread. For example, sleep-dependent memory consolidation is governed by reactivation, neocortical-thalamic- hippocampal dialogue, and synaptic downscaling (Diekelmann et al., 2009; Wilhelm et al., 2012; Goerke et al., 2017). Although there is some evidence of reactivation of emotional material during sleep (Cellini and Capuozzo, 2018; Hutchison et al., 2021), the neural patterns of activation and the specific electrophysiological frequencies that control autonomic and cognitive sleep-dependent emotion regulation remain unexplained (or, at best, are supported by conflicting strands of evidence). Second, in this review we focused on sleep-dependent modulation of emotional reactivity: however, many other forms of emotion-related processes may be modulated by sleep. These include extinction, habituation, mood, and intentional cognitive modulation of emotion. Future studies should state explicitly which kind of emotion-related process is being investigated; more broadly, the literature as a whole should seek to examine to what extent and in which ways each process is modulated by sleep.

## Data availability statement

The original contributions presented in the study are included in the article/Supplementary material, further inquiries can be directed to the corresponding author.

## Author contributions

GL and JM conceived the project idea and conducted the initial literature search. GL, HA, RL, JM, KT, DB, and MH screened and evaluated article titles, abstracts, and texts for inclusion in the review. HA reviewed a set of review articles and searching for articles that might be included in the review. BS conducted the statistical analyses. GL contributed to interpretation of those analyses. HA and DB conducted the narrative analysis. GL, JM, and KT prepared the manuscript for submission. All authors discussed the results and contributed to writing the manuscript. All authors contributed to the article and approved the submitted version.

## Funding

The study was funded by a National Research Foundation (NRF) Competitive Support for Unrated Research grant awarded to GL (grant number NRF116229).

## Conflict of interest

The authors declare that the research was conducted in the absence of any commercial or financial relationships that could be construed as a potential conflict of interest.

## Publisher's note

All claims expressed in this article are solely those of the authors and do not necessarily represent those of their affiliated organizations, or those of the publisher, the editors and the reviewers. Any product that may be evaluated in this article, or claim that may be made by its manufacturer, is not guaranteed or endorsed by the publisher.
