# Supplementary material for: Preferential consolidation of emotional reactivity during sleep: A systematic review and meta-analysis
Source: Front Behav Neurosci. 2022 Oct 4;16:976047. doi: 10.3389/fnbeh.2022.976047 (PMC9578377; doi:10.3389/fnbeh.2022.976047)
Supplement: Supplementary file 1 [file Data_Sheet_1.docx]

Supplementary Material

# Supplementary Data

## Study-by-study narrative reviews

Cunningham et al (2014) report a between-subjects study of 39 healthy participants, examining how physiological measures of reactivity (heart rate deceleration [HRD], skin conductance [SCR]) to negative and neutral images changed over a full night of sleep compared to a day of wakefulness. Participants viewed a set of 68 scenes that portrayed negatively arousing or neutral objects (34 of each valence) placed on plausible neutral backgrounds. Participants rated valence and arousal only at the initial encoding session, and were then called back for a recall task after 12 hrs. of either waking time or sleep, when HRD and SCR were measured. The sleep group had a significantly reduced heart rate deceleration and skin conductance response, to both negative and neutral stimuli, but there was no change in the wake group. The authors suggested this provided evidence for a general depotentiation of physiological reactivity to neutral and negative stimuli following sleep, but no change in visceral reactivity when the delay was spent awake.

Goldstein et al (2013) report a cross-over study involving 18 participants who performed an emotion-anticipation task using negative, neutral, and ambiguous stimuli inside an fMRI machine. They did this after a usual night's sleep and after 24 hrs. of monitored sleep deprivation. Subjective valence ratings were not reported. Findings indicate that following sleep deprivation, an enhanced anticipatory response was common across cue types in the amygdala, independence of valence, or certainty. This potentially reflects a preparatory brain state of heightened threat expectancy. The authors also found that trait anxiety confers vulnerability to the amplifying effects of sleep loss in the anterior insula.

Hot et al (2016) provide a preliminary report (conference abstract only) of 60 participants who watched a negative movie, in whom half were given no instructions, and half were instructed to use an emotional reappraisal strategy given by the experimenter. Each group was subdivided into a nap group and a wake group, after which non-emotional pictures from the movie were shown to all participants. Heart-rate deceleration (HRD) and emotional scales were measured before and after the movie, and before and after the neutral pictures. In the emotion regulation group who napped, there was an association with a decrease in negative feelings, but no difference in HRD was observed. In all the other groups, emotional feeling and HRD were similar.

Kuriyama et al (2013) describe a between-subjects study involving 62 healthy college students undertaking a 3-day memory experiment, divided into 4 sub-groups (total sleep deprivation and directed forgetting total sleep deprivation and directed remembering normal sleep and directed forgetting, and normal sleep and directed remembering). All subjects participated in a baseline trial looking at video clips of car crashes or safe driving to determine baseline emotional response; after Day 1, the sleep-deprived subjects were totally deprived of nocturnal sleep but then obtained recovery sleep on Day 2; the controls slept normally throughout. On day 3, all subjects participated in an event recognition task and skin conductance response (SCR) was measured at both baseline and subsequent tasks as a measure of implicit emotional reaction. Sleep deprivation reduced the SCR of the “directed to remember” group when viewing neutral or safe driving; in contrast, those who slept normally maintained the same level of SCL.

Lau et al (2020) report a between-subjects study of 66 healthy people, assigned to a wake condition, a 30-min nap condition (non-REM sleep), or a 90-min nap condition (with the aim of entering REM sleep). The study population also included depressed individuals undergoing the same experiment but for this review, we include only findings in healthy individuals. The volunteers undertook a validated emotional face perception task examining 4 emotions (happy, sad, fearful, and angry) at a range of intensity levels, before and after the nap or wake period. Ratings of mildly expressive fearful faces increased after wakefulness but remained stable in the 90-min-nap condition. There was an increased rating of fully expressive angry faces and mildly expressive sad faces in individuals after a 30-min non-REM nap, suggesting that there are potentially different effects of a non-REM nap on emotion perception, compared to a REM nap and remaining awake.

Minkel et al (2011) report a between-subjects study of 23 healthy subjects, who watched two film clips that were either sad or amusing. They were then randomized to either a night of sleep deprivation or a full night of sleep before watching another pair of sad and amusing clips. After each clip they self-rated their subjective emotional response; their reactions were filmed and analyzed for facial expressiveness. There was no statistical difference in the subjective emotional response to films, but sleep-deprived participants were significantly less expressive than the control participants who had slept. This suggested that sleep deprivation may have a larger effect on the behavioral indicators of emotion than on the subjective experience of emotion.

Reddy et al (2014) provide a preliminary report (conference abstract) of a between-subjects study of 42 adolescents aged between 13-17 yrs., randomized to either an idealized (9.5 hr.) sleep or a restricted sleep condition. At baseline and on the day following the sleep manipulation, the participants undertook tasks including an emotion regulatory task using the International Affective Picture System. The sleep-restricted group had evidence of decreased positive affectivity at the second assessment and demonstrated decreased emotion-regulatory ability when viewing negative images.

Reid et al (2019) provide a preliminary report (conference abstract) of a between-subjects randomized study of 51 healthy good sleepers, randomized to either one night of undisturbed sleep or one night of disrupted sleep with 8 forced awakenings. The participants undertook a task assessing attention bias towards threat-related vs neutral words before and after sleep: in the disruption of sleep group, there was no significant effect on attention bias for threat-related words. These findings indicate there was no clear change in attention bias before and after sleep in either group.

Schoch et al (2017) report a between-subjects study involving 228 subjects aged between 18-35 yrs., distributed across 4 experimental groups, each split into a sleep and wake group. One of the undertaken tasks involved looking at 72 neutral, negative, and positive images from the International Affective Picture Set, rating the valence and arousal of the pictures and then completing a free recall after a 12-hr retention interval of either sleep overnight or day-time wakefulness. Half of the participants also observed an interference set of images after a sleep or wake interval, which had equivalent valence ratings to the original set of images. Participants also rated these pictures. Additionally, half of the participants had an immediate retrieval task, meaning their memory strength was higher. In those groups, sleep benefited memory for emotional images but not for neutral images. However, participants without an immediate recall (and therefore a lower memory strength) showed no sleep benefit for either emotional or neutral images. Interestingly, comparing negative vs positively valenced pictures separately did not alter the general result pattern. After an interval of either sleep or waking, participants tended to rate the interference pictures as less negative but overall more arousing (irrespective of valence category). The authors argued that the strength of the memory trace before sleep and the sensitivity of the retrieval test after sleep are critical factors contributing to the detection of the benefit of sleep on memory for emotional and neutral stimuli.

Wagner et al (2002) report a between-subjects study of 24 male subjects aged 18-30 years, divided into two groups, and tested across 3-hr periods of early and late nocturnal sleep (sleep group) or corresponding intervals filled with wakefulness (wake group). They rated negative pictures from the International Affective Picture System before and after each sleep condition. Compared with early sleep and with the effects of the corresponding wake condition, late sleep (dominated by REM sleep) enhanced negative valence ratings of the pictures, whereas slow-wave-sleep early sleep enhanced positive valence ratings. The pictures were also rated as more arousing, and indicated a generally enhanced emotional reactivity, after sleep compared to wake periods.

## The Studies Included in the Quantitative Meta-analysis

Alfarra et al (2015) report a cross-over study comparing 10 sleep-deprived subjects aged 18-30 yrs. to a control group who were not sleep-deprived. They displayed positive, negative, and neutral pictures to the participants at baseline and then after the sleep condition, and measured response latencies, indicating reactivity to the emotional stimuli. The participants also completed a self-rating scale of valence for the images and electroencephalogram (EEG) event-related potential (ERP) data were acquired – specifically the late positive potential (LPP) component was studied (an established sensitive measure of attention to emotionally charged visual stimuli), as well as salivary cortisol measures. Relative to baseline testing, emotional pictures (negative or positive) were rated as less emotional following sleep deprivation. There was no significant change in cortisol concentrations or response latencies between groups. Prior to sleep deprivation, ERP responses to both emotionally positive and negative stimuli had significantly larger LLP amplitudes when compared to negative stimuli. However, after sleep deprivation the LPP response no longer discriminated between emotional (negative or positive) pictures or neutral, due to an increased amplitude response to the neutral pictures, suggesting that sleep loss interferes with the allocation of attention resources during an emotional task.

Ashton et al (2019) report data from 48 young adult participants who provided affective ratings for negative and neutral images whilst heart rate deceleration (HRD) and skin conductance responses (SCRs) were monitored. Following a 12-hr delay of sleep or wakefulness, participants completed an image recognition task where HRD, SCRs, and affective ratings were recorded again. SCRs were generally higher for negative rather than neutral images, but there were no significant differences between the two groups (sleep vs. wakefulness). HRD was generally stronger for negative images than neutral ones, and HRD responses to previously seen negative images were preserved oversleep but decreased over wakefulness. Changes in valence ratings for negative images increased (i.e. participants felt more positive about the images) over the 12-hr period, in both groups.

Baran et al (2012) report a between-subjects study of 106 young adults who rated negative and neutral pictures for valence and arousal during two sessions, separated either by 12 hrs. of daytime wakefulness or 12 hrs. including an overnight sleep. In the second session, participants completed a surprise recognition phase to see if they could distinguish previously seen pictures, as well as rating the valence and arousal. Negative pictures were rated with less negativity (more neutral) after a period of wakefulness compared to sleep: for neutral pictures, this effect was not seen. Similarly, arousal ratings for negative pictures were reduced over the wake period compared to the sleep group, although this did not reach statistical significance. As a period of waking was associated with attenuation of negative ratings compared to the maintenance of negative ratings during sleep, sleep may be protective of the emotional salience of a stimulus.

Bolinger et al (2018) report a crossover study of 16 healthy children aged 8-11 years who participated in both a sleep and a wake condition, with an interval of at least 12 days between each condition. They started with an encoding session where neutral and negative pictures were presented for the first time, followed by 10 hrs. of either sleep or wakefulness. They then had a recognition session, in which original pictures were presented alongside novel ones; valence and arousal ratings, HRD, and the late positive potential (LPP) of the electroencephalogram (EEG) were measured at each session. Sleep produced a less negative rating of negative images and a more negative valence of neutral images. Compared to the wake period, sleep led to a relative decrease in the LPP emotional response. In contrast to the subjective ratings and LPP, the HRD emotional response decreased across wake but not across sleep. The authors suggested that sleep decreases responses that are more subject to cognitive control while increasing responses that are generated more automatically.

Bolinger et al (2019) then reported a between-subjects study of 32 young adult participants, randomized to a sleep or wake group. An encoding session in which neutral and negative pictures were presented for the first time, was followed by 10 hrs. of either sleep or wakefulness. In a recognition session, original pictures were presented alongside novel ones, and valence and arousal ratings, HRD, and the LPP of the EEG were measured at each session. Sleeping after the first task (encoding) did not influence how valence or arousal ratings changed in the first 10 hrs. Sleeping after encoding, compared to staying awake, led to a relative decrease in LPP emotional response after 10 hrs. By contrast, a night of sleep (compared to a day awake) led to the preservation of the HRD aspect of the emotional response. This differed from a subsequent recognition task (data not extracted) at 10 days, which showed differing effects on valence ratings, HRD, and LPP which led the authors to suggest that subjective ratings (which are more susceptible to cognitive control) are influenced by sleep in the longer term.

Cellini et al (2016) report a between-subjects study of 46 healthy university students, who viewed a set of neutral, positive, and negative images before being randomized to a wake or a nap group. The nap was polysomnographically recorded and the group was further subdivided into a 90-min nap (non-REM) or a 120-min nap (to reach REM sleep). Participants were then exposed to the second set of emotional and neutral pictures before performing a recognition test. There were no significant differences in arousal or valence ratings of the pictures seen before and after sleep, whether REM or non-REM sleep.

Gujar et al (2011) report a between-subjects study of 36 healthy young adults, who performed an emotional face recognition task of 4 separate emotions (happiness, sadness, anger, fearfulness) and were then randomly assigned to a nap (90-min sleep opportunity, monitored polysomnographically) or no-nap group. The task was then repeated and the change in emotion reactivity was analyzed. There was a significant amplification of anger ratings in those in the wake group compared to the sleep group, as well as an amplification of fear, which was reduced in the nap group. In contrast to those negative emotions, there was a significant increase in the ratings of happy expressions after a sleep in the nap group. Further subdividing the nap group into those that reached REM sleep (*n*=8) and non-REM sleep (*n*=10) indicates that the REM group had a significant reduction in fear ratings and a significant increase in happy ratings, when compared to the non-REM group.

Jones et al (2018) report a between-subjects study comparing 40 young adults and 41 middle-aged adults, who viewed negative and neutral pictures and underwent a recognition test after a sleep or wake period. Emotional reactivity was also measured using valence reactivity and arousal reactivity. The increase in the valence of negative pictures tended to be smaller in the sleep group than the wake group in young adults but was similar between sleep and wake groups in middle-aged adults. Sleep appeared to preserve valence reactivity to negative pictures in young adults but not middle-aged adults, while the effect of sleep on reactivity for neutral pictures was similar between age groups.

Kuriyama et al (2010) report a between-subjects study of 28 healthy college students randomly assigned to a sleep deprivation or normal sleep (control) group. Both groups watched 14 movies, half of which depicted safe driving and half depicted a motor vehicle accident, in random order; and rated the fear they felt as well as measured the SCR. Participants then were either sleep-deprived for one night or allowed normal sleep and repeated the task on days 3 and 10. Sleep deprivation diminished the self-rated fear rating towards safe images, compared to the normal sleep group. Interestingly, physiological response (measured via SCR) was not significantly correlated to self-reported fear response ratings in either group.

Lipinska et al (2019) report a crossover study of 20 healthy women (and 21 women with Post-Traumatic Stress Disorder (PTSD), and 19 trauma-exposed but non-PTSD individuals: not within the scope of this review). The task included exposure to negatively valenced, positively valenced, and neutral pictures; measures of autonomic arousal in response to each picture were measured using heart rate (HR), pre-ejection period PEP (the interval from left ventricular depolarization to the opening of the aortic valve), ventricular ejection time (LVET) and skin conductance level (SCL). Initially, the participants performed the task before and after an 8-hr sleep, measured polysomnographically. On another day, they completed the task with an 8-hr waking interval. There was a substantial decrease in PEP sympathetic activation across the sleep-filled delay, and a small increase across the wake-filled delay. The authors suggest that whereas sleep was associated with maintained or decreased sympathetic activation, waking was associated with an increase in such activity.

Pace-Schott et al (2011) report a between-subjects study of 43 young adults who viewed negatively valenced or emotionally neutral pictures for two, afternoon sessions separated by 2.5 hrs. (participants were randomly allocated to a nap or wake condition). For each stimulus presentation, evoked SCR, HRD, and Corrugator supercilii EMG response (EMG) was measured, as well as subjective ratings of valence and arousal. During the second session, novel pictures were presented alongside previously seen images. Subjective valence and arousal ratings were not significantly different between the groups: however, for previously seen images, nap subjects demonstrated greater inter-session habituation in SCR and EMG (an effect that was greater in negatively valenced stimuli) and a trend toward lesser inter-session habituation in HRD. The authors, therefore, suggested that physiological but not subjective habituation to aversive images was enhanced by a daytime nap.

Prehn-Kristensen et al (2016) describe a crossover study of 16 healthy male children (and 16 male children diagnosed with mental disorders, not in the scope of this review). The boys completed a task rating the emotional content of pictures of facial expressions, then after 12 hrs. (of normal night-time sleep, or a day awake), they rated the emotional content again and completed a recognition task. Each participant took part in a sleep and wake condition; eye-tracking and pupillometry measurements were also recorded. There was an increase in pupil dilatation in response to previously seen faces in the sleep condition but not the wake condition; this was not statistically significant for the different emotions of anger, fear, and happiness. There were no significant differences in the ratings of intensity in the sleep or wake condition. The authors suggested there were no signs of sleep-related emotional regulation in the children in this study.

Tempesta et al (2010) report a study of 40 female university students who rated pleasant, neutral, and unpleasant pictures for valence and arousal. They were tested before and after either a night of sleep deprivation or normal sleep (control group) for one night. No significant effect of sleep deprivation was observed on the ratings of pleasant or unpleasant pictures. However, sleep-deprived participants perceived the neutral images more negatively compared to the well-rested control group. They also rated unpleasant (negative) images as significantly more arousing than pleasant ones.

Tempesta et al (2015) report a subsequent study of 75 subjects divided into poor sleeper, good sleeper, and sleep deprivation groups. The participants viewed positive, negative, and neutral images and rated valence and arousal at an initial session, a second session after an hour, and the third session after one night of normal sleep or sleep deprivation. Sleep-deprived subjects rated positive and neutral pictures more negatively compared to well-rested subjects. There was no difference in the valence between negative pictures, and no difference in the arousal ratings between the groups for positive, negative, or neutral pictures.

# Supplementary Figures and Tables

## Supplementary Figures

### Risk of Bias: All Studies


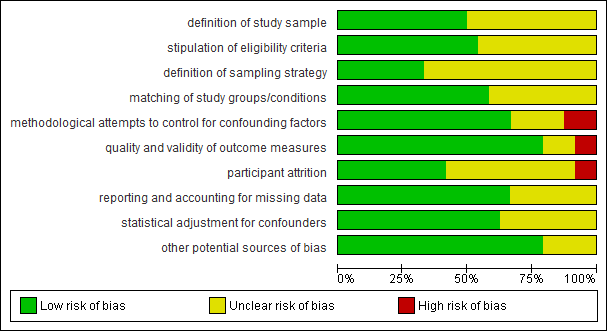


**Supplementary Figure 1.** Percentage Of All Reviewed Studies Rates as Being of Low, Unclear, And High Risk of Bias on Each Rated Methodological Dimension (N = 24)

### Risk of Bias: Each Study


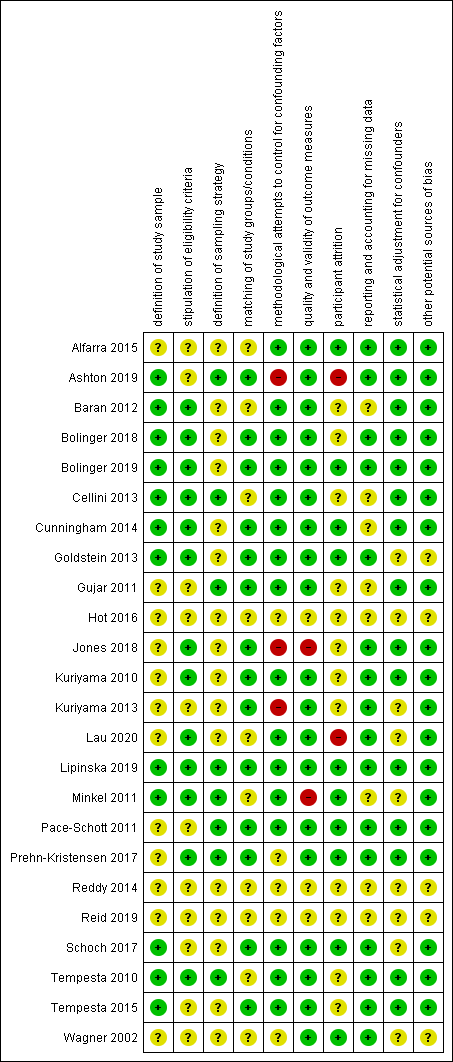


**Supplementary Figure 2.** Risk Of Bias (Low, Unclear, Or High) For Each Reviewed Study on Each Rated Methodological Dimension (N = 24

### Metanalysis Results: Heart Rate Deceleration (HRD)

#### Physiological measures

##### Negative Sleep versus Negative Comparator

If we include the HRD studies only, the results are more consistent and the results are suggestive of a small to moderate effect but not statistically significant (*p*=0.10), perhaps due in part to the small sample size of only 62 participants.

**Supplementary Figure 3.** Comparison of studies assessing HRD in response to negative stimuli across groups

### Neutral Sleep versus Neutral Comparator

If we restrict it to the 3 studies and 62 participants where the outcome was HRD, again the results suggest a small moderate effect size of -0.05 (95% CI -0.42, 0.33) which is not statistically significant at *p*=0.80 (see Supplementary Figure 2).

**.** **Supplementary Figure 4.** Comparison of studies assessing HRD in response to neutral stimuli across groups

# Subgroup analyses

## Valence

#### Type of control

There was no statistically significant subgroup effect of the type of control condition (p=0.78 for negative and 0.64 for neutral comparisons) but there is for positive (p=0.01)

##### Negative Sleep Versus Negative Comparator

**Supplementary Figure 5.** Comparison of the subgroup effect of the type of control used on valence in response to negative stimuli

##### Neutral Sleep Versus Neutral Comparator

**Supplementary Figure 6.** Comparison of the subgroup effect of the type of control used on valence in response to neutral stimuli

##### Positive Sleep Versus Positive Comparator

**Supplementary Figure 7.** Comparison of the subgroup effect of the type of control used on valence in response to positive stimuli

#### Nap or whole night

There were no statistically significant subgroup effects based on nap/whole night sleep for the neutral (p=0.09) comparison but there was for the negative comparison (p=0.04) and the positive comparison (p=0.02)

##### Negative Sleep Versus Negative Comparator

**Supplementary Figure 8.** Comparison of the subgroup effect of the sleep condition used on valence in response to negative stimuli

##### Neutral Sleep Versus Neutral Comparator

**Supplementary Figure 9.** Comparison of the subgroup effect of the sleep condition used on valence in response to neutral stimuli

##### Positive Sleep Versus Positive Comparator

**Supplementary Figure 10.** Comparison of the subgroup effect of the sleep condition used on valence in response to positive stimuli

#### IAPS

There were no significant subgroup effects based on whether or not the IAPS was used for the negative comparison (p=0.42), the neutral comparison (p=0.26) or the positive comparison (p=0.17)

##### Negative Sleep Versus Negative Comparator

**Supplementary Figure 11.** Comparison of the subgroup effect of the IAPS on valence in response to negative stimuli

##### Neutral Sleep Versus Neutral Comparator

**Supplementary Figure 12.** Comparison of the subgroup effect of the IAPS on valence in response to neutral stimuli

##### Positive Sleep Versus Positive Comparator

**Supplementary Figure 13.** Comparison of the subgroup effect of the IAPS on valence in response to positive stimuli

#### REM

There are no statistically significant subgroup effects in the negative (p=0.92) comparison. However, in the neutral comparison there is a significant effect in the non-REM studies that is not seen in the REM studies (p=0.04). Similarly there was a subgroup effect in the positive comparison (p=0.01)

##### Negative Sleep Versus Negative Comparator

**Supplementary Figure 14.** Comparison of the subgroup effect of REM sleep on valence in response to negative stimuli

##### Neutral Sleep Versus Neutral Comparator

**Supplementary Figure 15.** Comparison of the subgroup effect of REM sleep on valence in response to neutral stimuli

##### Positive Sleep Versus Positive Comparator

**Supplementary Figure 16.** Comparison of the subgroup effect of REM sleep on valence in response to positive stimuli

## Arousal

### Type of control

There were no statistically significant subgroup effects based on the type of control for the negative (p=0.39) or the neutral (p=0.66) comparisons

#### Negative Sleep Versus Negative Comparator

**Supplementary Figure 17.** Comparison of the subgroup effect of the type of control used on arousal in response to negative stimuli

#### Neutral Sleep Versus Neutral Comparator

**Supplementary Figure 18.** Comparison of the subgroup effect of the type of control used on arousal in response to neutral stimuli

### Nap/Full night

There were no statistically significant subgroup effects in the negative comparison (p=0.66) or the neutral comparison (p=0.37)

#### Negative Sleep Versus Negative Comparator

**Supplementary Figure 19.** Comparison of the subgroup effect of the sleep condition used on arousal in response to negative stimuli

#### Neutral Sleep Versus Neutral Comparator

**Supplementary Figure 20.** Comparison of the subgroup effect of the sleep condition used on arousal in response to neutral stimuli

### IAPS

All studies used the IAPS

### REM

There were no statistically significant subgroup effects in the negative (p=0.39) or neutral (p=0.66) comparisons.

#### Negative Sleep Versus Negative Comparator

**Supplementary Figure 21.** Comparison of the subgroup effect of REM sleep on arousal in response to negative stimuli

#### Neutral Sleep Versus Neutral Comparator


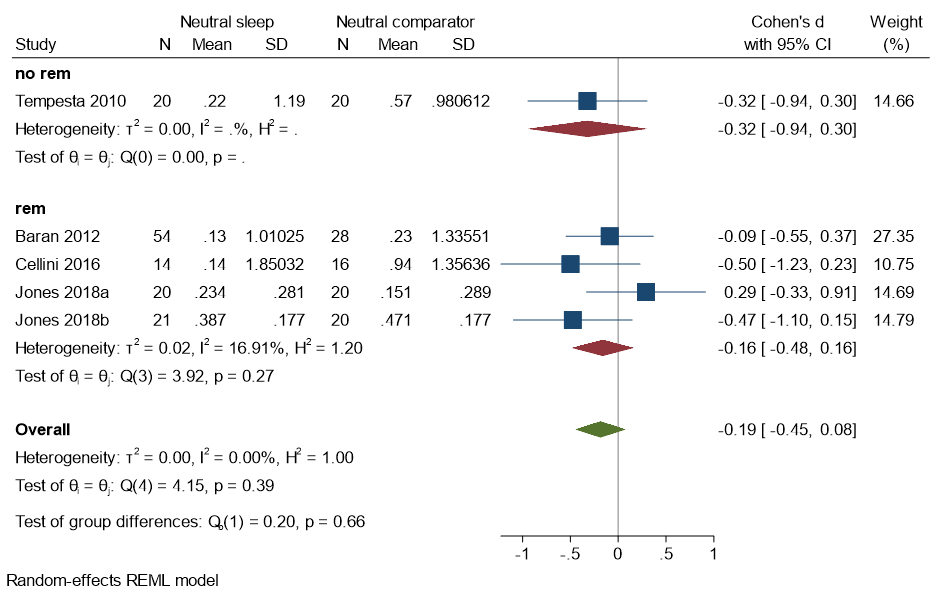


**Supplementary Figure 22.** Comparison of the subgroup effect of REM sleep on arousal in response to neutral stimuli

## Publication Bias

### Funnel Plots

####
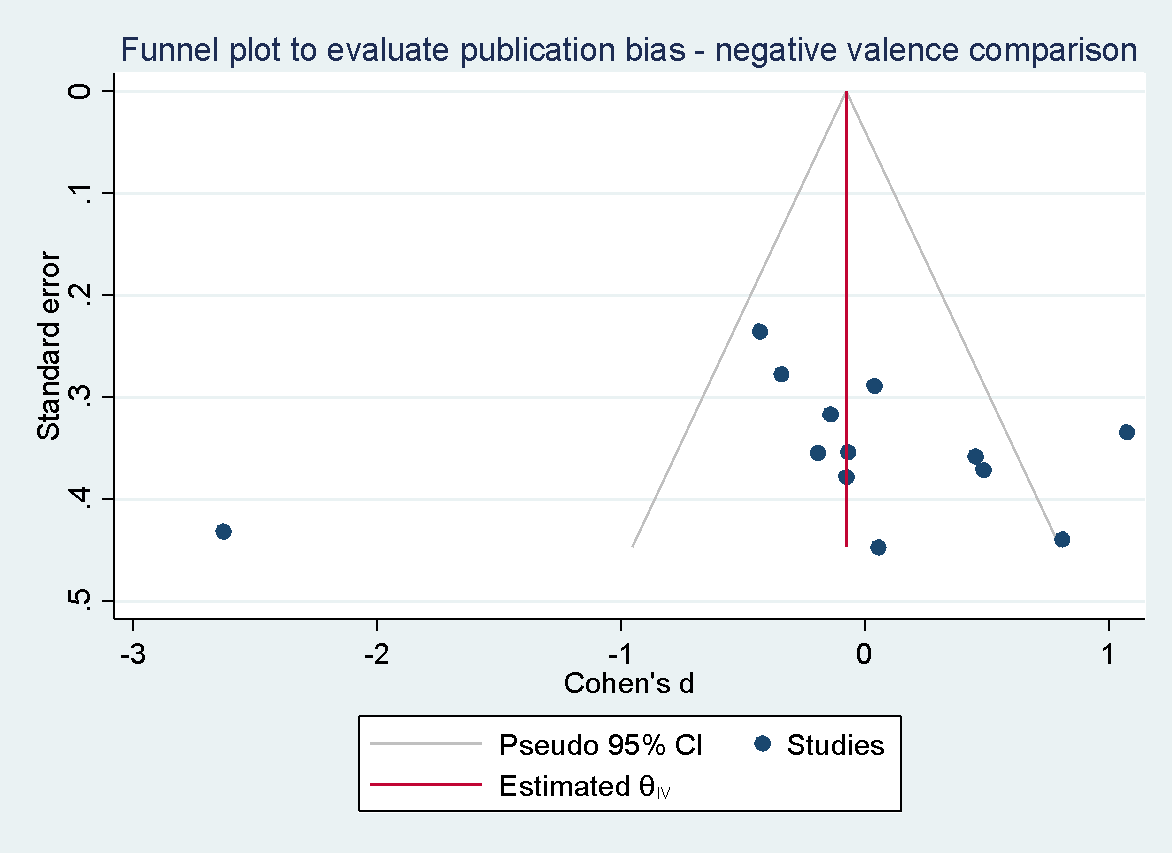
Negative Valence Comparison

**Supplementary Figure 23.** Publication bias assessment of self-reported valence in responses to negative stimuli

#### Neutral Valence Comparisons


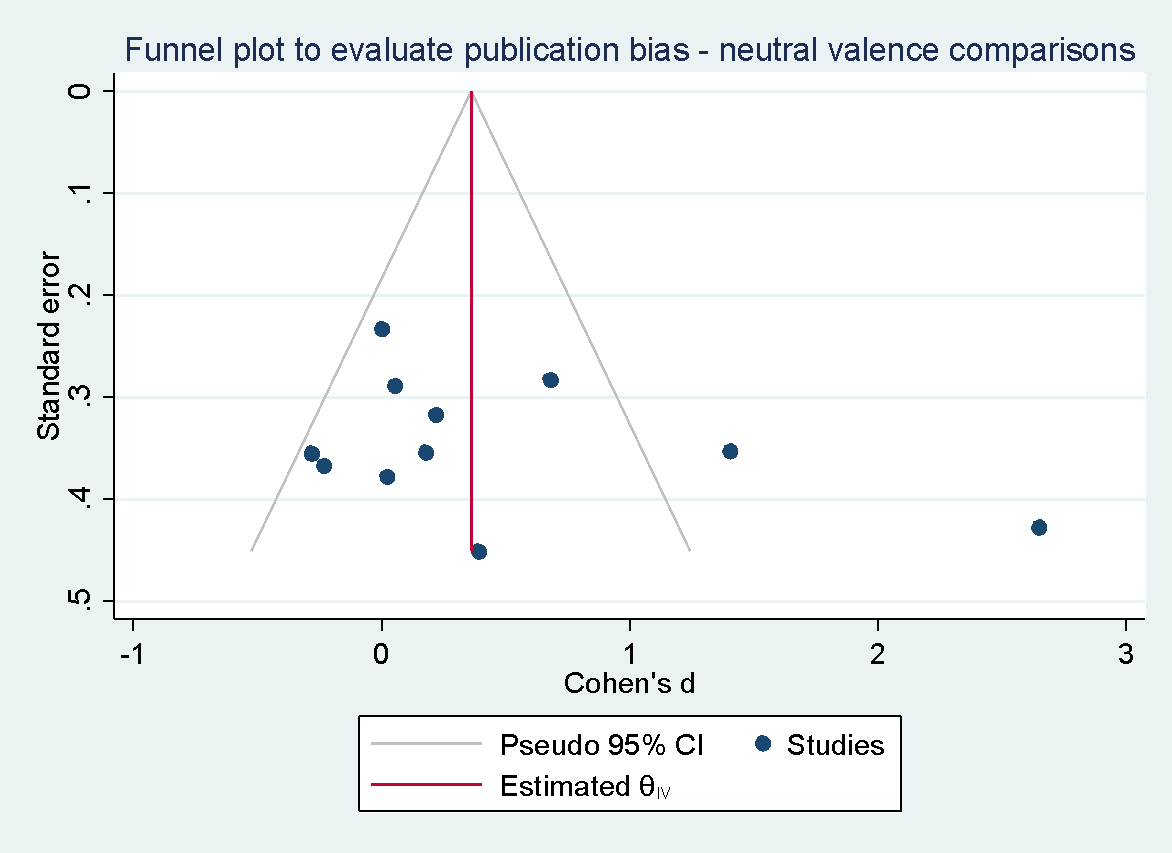


**Supplementary Figure 24.** Publication bias assessment of self-reported valence in responses neutral stimuli
